# Corrigendum to ‘Primary percutaneous stenting for palliative biliary drainage of patients with malignant hilar biliary obstruction: TESLA trial’ [JHEP Reports 7 (2025) 101541]

**DOI:** 10.1016/j.jhepr.2025.101633

**Published:** 2025-11-04

**Authors:** Stijn Franssen, Merve Rousian, Victorien van Verschuer, Marco Bruno, Michail Doukas, Lydi van Driel, Marjolein Homs, Behnam Mohseny, Roeland de Wilde, Jeroen de Jonge, Wojciech Polak, Robert Porte, Diederik Bijdevaate, Adriaan Moelker, Bas Groot Koerkamp

**Affiliations:** 1 Department of Surgery, Erasmus MC Cancer Institute, Rotterdam, The Netherlands; 2 Department of Gastroenterology and Hepatology, Erasmus MC Cancer Institute, Rotterdam, The Netherlands; 3 Department of Pathology, Erasmus MC Cancer Institute, Rotterdam, The Netherlands; 4 Department of Medical Oncology, Erasmus MC Cancer Institute, Rotterdam, The Netherlands; 5 Department of Radiology, Erasmus MC Cancer Institute, Rotterdam, The Netherlands

It has come to our attention that the published version of our article contains the following errors and omissions:

The acknowledgement for Dr. Karen T. Brown was inadvertently omitted. The authors wish to add the following acknowledgement: “The authors wish to express their sincere gratitude to Dr. Karen T. Brown, Interventional Radiologist at Weill Cornell Medicine, New York, for her guidance and support throughout this project.” This acknowledgement has now been included in the updated online version of the manuscript.

In the graphical abstract, highlights, and concluding discussion, the percentage of patients who commenced palliative treatment was incorrectly reported as 61.2%. The correct value is 62.7%, consistent with Table 2 (page 4), the Results section (page 5), and the beginning of the Discussion (page 5). Accordingly, the text in the highlights (page 1) and end of the discussion (page 7) has been updated to reflect the correct value of 62.7%. The graphical abstract has also been corrected.

The authors apologize for these oversights. These corrections do not affect the overall conclusions of the study.

