# Supplementary material for: Primary percutaneous stenting for palliative biliary drainage of patients with malignant hilar biliary obstruction: TESLA trial
Source: JHEP Rep. 2025 Sep 11;7(11):101541. doi: 10.1016/j.jhepr.2025.101541 (PMC12529366; doi:10.1016/j.jhepr.2025.101541)
Supplement: Multimedia component 1 [file mmc1.pdf]

Primary percutaneous stenting for palliative biliary drainage of patients with malignant hilar biliary obstruction: TESLA trial☆

Stijn Franssen, Merve Rousian, Victorien van Verschuer, Marco Bruno, Michail Doukas, Lydi van Driel, Marjolein Homs, Behnam Mohseny, Roeland de Wilde, Jeroen de Jonge, Wojciech Polak, Robert Porte, Diederik Bijdevaate, Adriaan Moelker, Bas Groot Koerkamp

Table of contents

Supplementary methods……………………………………………………………….2

Fig. S1…………………………………………………………………………………3

Table S1……………………………………………………………………………… 4

Supplementary methods. Detailed description of study intervention.

The percutaneous transhepatic bile duct puncture was performed with an 18G or 21G Chiba needle. Then, a 4 or 5 Fr sheath (e.g., Neffset, Cook Medical, Bloomington, IN)) was placed and a 0.032-0.038 inch guidewire with 4 Fr catheter was advanced beyond the bile duct stricture or obstruction without passing the ampulla. After crossing the stricture, the guidewire was preferably replaced by a stiff Amplatz guidewire. The sheath was replaced by a 7 or 8 Fr sheath allowing for a brush and stent placement. Brush cytology was performed by moving the brush five times back and forth through the stricture. This was repeated with a second brush to increase the diagnostic yield. Before stent insertion, the stricture was dilated with a balloon catheter that was pushed over the guidewire (diameter 6 to 8 mm). Next, a biliary stent (Hilzo biliary stent, uncovered straight type, 8 or10 mm diameter and 6 or 8 cm length) was inserted, again without advancing the wire beyond the ampulla. The puncture tract was sealed with 0.5 gr Avitene (Becton Dickinson NJ, USA) mixed with 8 ml of iodine contrast agent to visualize the sealing track. No external biliary drain was left after stent placement. Crossing of the ampulla with a drain or stent (i.e., internal drainage) was avoided.

When multiple stents were placed, a guidewire was inserted in 2 or 3 segments before balloon dilatation and stent placement. Multiple stents were mostly placed in a "kissing" fashion (i.e., adjacent in the common hepatic bile duct), rather than in a fenestrated fashion. Advancing the guidewire beyond the tumor can be challenging and may require several attempts. Whenever the tumor could not be passed during the procedure, an external biliary drain was placed. A second attempt of stenting was performed after 4 to 7 days.

All patients received antibiotics before and after biliary drainage. All patients received a one-time preprocedural intravenous dose of cefuroxime 1500mg and metronidazole 500mg and 5 days of oral amoxicillin/clavulanic acid 3dd 625mg after the procedure, or ciprofloxacin 2dd 500mg in patients with a penicillin allergy. Patients who presented with a bilirubin above 15.0 mg/dL were already started on the same oral antibiotics prior to the procedure to minimize the risk of spontaneous cholangitis before biliary drainage.

Fig. S1. Serum bilirubin level curve of all patients in the first 28 days after PPS.

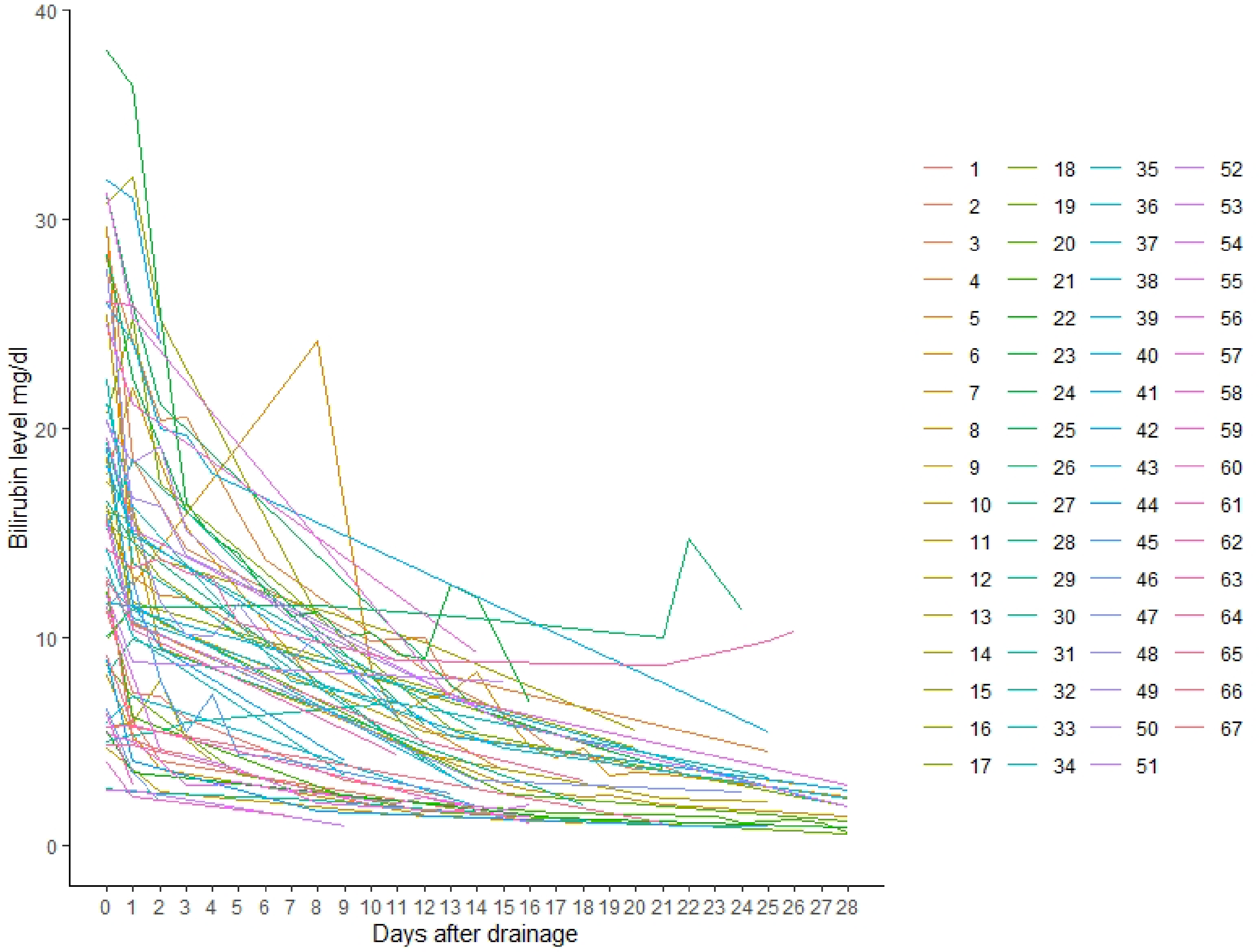

Table S1. Definitions of severe drainage-related complications.

Complication	Criteria
Cholangitis	Elevation in temperature ≥ 38,5°C and Leukocytes ≥10 *10^9/L, thought to have a biliary cause and requiring invasive intervention, without concomitant evidence of acute cholecystitis.
Acute cholecystitis	Radiologic evidence of cholecystitis, elevation in temperature more than 38.5°C and Leukocytes ≥10 *10^9/L, requiring emergency cholecystectomy or percutaneous drainage.
Persistent jaundice	Persistent elevated bilirubin levels after therapeutic success had initially been obtained, without signs of cholangitis or cholecystitis, requiring a reintervention.
Acute pancreatitis	Two or more of the following criteria be met for the diagnosis of acute pancreatitis: abdominal pain suggestive of pancreatitis, serum amylase or lipase level greater than three times the upper normal value, or characteristic imaging findings.
Biliary leak	Symptomatic intra-abdominal bile leakage due to bile duct perforation or leakage from the puncture tract documented by any radiographic technique requiring intervention.
Hemorrhage	Clinical evidence of bleeding requiring blood transfusion or reintervention.
