# Supplementary material for: Primary percutaneous stenting for palliative biliary drainage of patients with malignant hilar biliary obstruction: TESLA trial
Source: JHEP Rep. 2025 Sep 11;7(11):101541. doi: 10.1016/j.jhepr.2025.101541 (PMC12529366; doi:10.1016/j.jhepr.2025.101541)
Supplement: Multimedia component 2 [file mmc2.pdf]

**Primary percutaneous placement of metal stents for palliative biliary drainage in patients with a primary malignant perihilar stricture**

# RESEARCH PROTOCOL

**(July 2022)**

**Principal Investigator:**
B. Groot Koerkamp, MD, PhD; Erasmus MC

**Study Sponsor:**
Prof. dr. J.M. Hendriks, MD, PhD; Erasmus MC

**Study Coordinator:**
S. Franssen, MD; Erasmus MC

**PROTOCOL TITLE 'Primary percutaneous placement of metal stents for palliative biliary drainage in patients with a primary malignant perihilar stricture**

| | |
|---|---|
| **Protocol ID** | **NL71124.078.19** |
| **Short title** | **TESLA** |
| **EudraCT number** | **Not applicable.** |
| **Version** | **5.0** |
| **Date** | **29-07-2022** |
| **Coordinating investigator/project leader** | **S. Franssen, MD**<br>s.franssen@erasmusmc.nl<br>Erasmus MC, University Medical Center<br>Department of Surgery<br>PO BOX 2040<br>3000 CA Rotterdam, The Netherlands |
| **Principal investigator(s)** | **Erasmus MC, Rotterdam**<br>B. Groot Koerkamp, MD, PhD<br>b.grootkoerkamp@erasmusmc.nl<br>Erasmus MC, University Medical Center<br>Department of Surgery<br>PO BOX 2040, 3000 CA Rotterdam<br><br>**UMC Utrecht, Utrecht**<br>J. Hagendoorn, MD, PhD<br>j.hagendoorn-3@umcutrecht.nl<br>Department of Surgery<br>PO BOX 85500, 3584 CX Utrecht<br><br>**Amsterdam University Medical Center (both locations AMC and VUmc), Amsterdam**<br>J.I. Erdmann, MD, PhD,<br>j.i.erdmann@amsterdamumc.nl<br>Department of Surgery |

| | |
|---|---|
| | PO BOX 7057, 1007 MB, Amsterdam<br><br>**Oslo University Hospital (Rikshospitalet), Oslo, Norway**<br>K. Lassen, MD, PhD<br>krlass@ous-hf.no<br>Department of Surgery<br>PO Box 4950, N-0424 Oslo |
| **Sponsor** | **Erasmus MC, Rotterdam**<br>Prof. dr. J.M. Hendriks, MD, PhD, Head of Department of Surgery<br>Dr Molewaterplein 40<br>3015 GD Rotterdam, The Netherlands |
| **Co-investigators**<br><br>***Multicenter research: per site*** | <br><br>**Erasmus MC, Rotterdam, The Netherlands**<br>A. Moelker, MD, PhD, Department of Interventional radiology<br>D. Bijdevaate, MD, PhD, Department of Interventional Radiology<br>Prof. dr. J.N.M. IJzermans, MD, PhD, Department of Surgery<br>L.M.J.W. van Driel, MD, PhD, Department of Gastroenterology and liver disorders<br>L.G.M. Bode, MD, PhD, Department of Infectious Diseases<br><br>**UMC Utrecht, Utrecht**<br>Prof. dr. I.H.M. Borel Rinkes, MD, PhD, Department of Surgery<br>M.L.J. Smits, MD, PhD, Department of Interventional Radiology<br>K.P. Van Lienden, MD, PhD, Department of Interventional Radiology<br><br>**Amsterdam UMC (both locations AMC and VUmc), Amsterdam** |

| | |
|---|---|
| | Dr. IJ.A.J. Zijlstra, MD, PhD, Department of Interventional Radiology<br><br>Prof. dr. O.M. van Delden, MD, PhD, Department of Interventional Radiology<br><br>Dr. R.P. Voermans, MD, PhD, Department of Gastroenterology and liver disorders<br><br>**Oslo University Hospital (Rikshospitalet), Oslo**<br>Dr. E. J. Dorenberg, MD, PhD, Department of Interventional Radiology |
| **Subsidising party** | **Not applicable** |
| **Independent expert (s)** | **S.M. Lagarde, MD, PhD**<br><br>s.lagarde@erasmusmc.nl<br><br>Erasmus MC, University Medical Center<br><br>Department of Surgery<br><br>PO BOX 2040<br><br>3000 CA Rotterdam, The Netherlands |

## PROTOCOL SIGNATURE SHEET

| Name | Signature | Date |
|---|---|---|
| **Sponsor or legal representative:**<br><br>**Erasmus MC, University Medical Center Head of Department of Surgery:**<br><br>Prof. J.M. Hendriks, MD, PhD | | |
| **Principal Investigator:**<br><br>B. Groot Koerkamp, MD, PhD, surgeon | | |

## PROTOCOL SIGNATURE SHEET

| Name | Signature | Date |
|---|---|---|
| **Sponsor or legal representative:**<br><br>**Erasmus MC, University Medical Center Head of Department of Surgery:**<br><br>Prof. J.M. Hendriks, MD, PhD | | 11/08/22 |
| **Principal Investigator:**<br><br>B. Groot Koerkamp, MD, PhD, surgeon | | 29/7/'22 |

**TABLE OF CONTENTS**


1. INTRODUCTION AND RATIONALE ........ 12
2. OBJECTIVES ........ 14
3. STUDY DESIGN ........ 15
4. STUDY POPULATION ........ 17
   - 4.1 Population (base) ........ 17
   - 4.2 Inclusion criteria ........ 17
   - 4.3 Exclusion criteria ........ 17
   - 4.4 Sample size calculation ........ 18
5. TREATMENT OF SUBJECTS ........ 19
   - 5.1 Investigational product/treatment ........ 19
   - 5.2 Use of co-intervention (if applicable) ........ 20
   - 5.3 Escape medication (if applicable) ........ 20
6. INVESTIGATIONAL PRODUCT ........ 21
   - 6.1 Name and description of investigational product(s) ........ 21
   - 6.2 Summary of findings from non-clinical studies ........ 21
   - 6.3 Summary of findings from clinical studies ........ 21
   - 6.4 Summary of known and potential risks and benefits ........ 21
   - 6.5 Description and justification of route of administration and dosage ........ 21
   - 6.6 Dosages, dosage modifications and method of administration ........ 21
   - 6.7 Preparation and labelling of Investigational Medicinal Product ........ 21
   - 6.8 Drug accountability ........ 21
7. NON-INVESTIGATIONAL PRODUCT ........ 22
8. METHODS ........ 23
   - 8.1 Study parameters/endpoints ........ 23
     - 8.1.1 Main study parameter/endpoint ........ 23
     - 8.1.2 Secondary study parameters/endpoints ........ 23
     - 8.1.3 Other study parameters (if applicable) ........ 23
   - 8.2 Randomisation, blinding and treatment allocation ........ 23
   - 8.3 Study procedures ........ 23
   - 8.4 Withdrawal of individual subjects ........ 25
     - 8.4.1 Specific criteria for withdrawal (if applicable) ........ 25
   - 8.5 Replacement of individual subjects after withdrawal ........ 25
   - 8.6 Follow-up of subjects withdrawn from treatment ........ 25
   - 8.7 Premature termination of the study ........ 25
9. SAFETY REPORTING ........ 27
   - 9.1 Temporary halt for reasons of subject safety ........ 27
   - 9.2 AEs, SAEs and SUSARs ........ 27
     - 9.2.1 Adverse events (AEs) ........ 27
     - 9.2.2 Serious adverse events (SAEs) ........ 27
     - 9.2.3 Suspected unexpected serious adverse reactions (SUSARs) ........ 28
   - 9.3 Annual safety report ........ 28
   - 9.4 Follow-up of adverse events ........ 28

9.5 Data Safety Monitoring Board (DSMB) .... 28
10. STATISTICAL ANALYSIS .... 29
10.1 Primary study parameter(s) .... 29
10.2 Secondary study parameter(s) .... 29
10.3 Other study parameters .... 30
10.4 Interim analysis (if applicable) .... 30
11. ETHICAL CONSIDERATIONS .... 31
11.1 Regulation statement .... 31
11.2 Recruitment and consent .... 31
11.3 Objection by minors or incapacitated subjects (if applicable) .... 31
11.4 Benefits and risks assessment, group relatedness .... 31
11.5 Compensation for injury .... 32
11.6 Incentives (if applicable) .... 32
12. ADMINISTRATIVE ASPECTS, MONITORING AND PUBLICATION .... 33
12.1 Handling and storage of data and documents .... 33
12.2 Monitoring and Quality Assurance .... 34
12.3 Amendments .... 34
12.4 Annual progress report .... 34
12.5 Temporary halt and (prematurely) end of study report .... 34
12.6 Public disclosure and publication policy .... 35
13. STRUCTURED RISK ANALYSIS .... 36
13.1 Potential issues of concern .... 36
13.2 Synthesis .... 36
14. REFERENCES .... 37
15. APPENDICES .... 38

## LIST OF ABBREVIATIONS AND RELEVANT DEFINITIONS

| | |
|---|---|
| **ABR** | **General Assessment and Registration form (ABR form), the application form that is required for submission to the accredited Ethics Committee; in Dutch: Algemeen Beoordelings- en Registratieformulier (ABR-formulier)** |
| **AE** | **Adverse Event** |
| **AR** | **Adverse Reaction** |
| **CA** | **Competent Authority** |
| **CCMO** | **Central Committee on Research Involving Human Subjects; in Dutch: Centrale Commissie Mensgebonden Onderzoek** |
| **CNS** | **central nervous system** |
| **CV** | **Curriculum Vitae** |
| **DSMB** | **Data Safety Monitoring Board** |
| **ERCP** | **Endoscopic Retrograde Cholangio-Pancreatography** |
| **EU** | **European Union** |
| **EudraCT** | **European drug regulatory affairs Clinical Trials** |
| **GCP** | **Good Clinical Practice** |
| **GDPR** | **General Data Protection Regulation; in Dutch: Algemene Verordening Gegevensbescherming (AVG)** |
| **IB** | **Investigator’s Brochure** |
| **IC** | **Informed Consent** |
| **IMP** | **Investigational Medicinal Product** |
| **IMPD** | **Investigational Medicinal Product Dossier** |
| **METC** | **Medical research ethics committee (MREC); in Dutch: medisch-ethische toetsingscommissie (METC)** |
| **pCCA** | **Perihilar Cholangiocarcinoma** |
| **(S)AE** | **(Serious) Adverse Event** |
| **SEMS** | **Self-Expandable Metal Stent** |
| **SPC** | **Summary of Product Characteristics; in Dutch: officiële productinformatie IB1-tekst** |
| **Sponsor** | **The sponsor is the party that commissions the organisation or performance of the research, for example a pharmaceutical company, academic hospital, scientific organisation or investigator. A party that provides funding for a study but does not commission it is not regarded as the sponsor, but referred to as a subsidising party.** |
| **SUSAR** | **Suspected Unexpected Serious Adverse Reaction** |

| | |
|---|---|
| **UAVG** | **Dutch Act on Implementation of the General Data Protection Regulation; in Dutch: Uitvoeringswet Algemene verordening gegevensbescherming** |
| **WMO** | **Medical Research Involving Human Subjects Act; in Dutch: Wet Medisch-wetenschappelijk Onderzoek met Mensen** |

## SUMMARY

**Rationale:** Most patients with perihilar cholangiocarcinoma (pCCA) are ineligible for curative-intent resection because of metastatic disease, locally advanced disease, or due to comorbidity. The key to successful palliative treatment is adequate biliary drainage to improve the patient's wellbeing and to allow for palliative systemic therapy. Endoscopic biliary drainage with plastic stents is the most common technique in the Netherlands. The main problem of this approach is that the stents cause bacterial colonization of the previously sterile intrahepatic bile ducts, because the stents cross the ampulla. Cholangitis often develops, especially if undrained segments become colonized. This is reflected by a 35% mortality within 3 months after diagnosis in patients who are ineligible for curative-intent resection. Most of these patients die from biliary obstruction and cholangitis without known metastatic disease. The only method to avoid colonization of the bile ducts is percutaneous placement of uncovered self-expandable metal stents (SEMS) that do not cross the ampulla.

**Objective**: To proof safety and effectiveness of direct percutaneous SEMS placement for palliative treatment of primary malignant perihilar stricture.

**Study design:** We aimed to perform a proof-of-concept pilot study at Erasmus MC , including 10 patients. The expected inclusion period was 1 year. Now all patients have been included in the pilot cohort and feasibility and safety has been determined, an expansion cohort will be added which includes UMC Utrecht, Amsterdam UMC and Oslo University Hospital (Norway), as participating centers in order to perform a multicenter phase-II trial. The aim is to assess the effectiveness and further assess safety of direct SEMS placement for palliative treatment of a primary malignant perihilar stricture. In total 67 patients will be included and analyzed in this study.

**Study population:** Patients with unresectable primary malignant perihilar obstruction on imaging with histopathological confirmation or high clinical suspicion (as determined by the multidisciplinary hepatobiliary team) who did not undergo previous endoscopic or percutaneous drainage procedures and who have no signs of cholangitis.

**Intervention**: Percutaneous transhepatic biliary drainage, by bridging significant ductal obstruction by self-expandable fenestrated metal stents without cannulation of the ampulla.

**Main study parameters/endpoints:** 6-month overall survival (OS), stent-related complications according to Clavien-Dindo grading system within 90 days (see Table 2.), absolute and relative

(%) bilirubin decrease after 14 days, the number of scheduled and unscheduled reinterventions within 90 days and quality of life.

**Nature and extent of the burden and risks associated with participation, benefit and group relatedness:** The percutaneous intervention is an alternative approach to the standard of care, which is endoscopic biliary drainage. Complications due to the transhepatic biliary drainage, i.e. bleeding, infection and bile leakage are uncommon (<5%).
We hypothesize that direct SEMS placement in patients with unresectable primary malignant perihilar obstruction minimizes post-drainage cholangitis and mortality, requires fewer reinterventions, and increases the rate of patients receiving palliative chemotherapy.
In this study, patients will undergo an invasive procedure with hospital discharge after a few days. This corresponds to the standard of care. Follow-up appointments are planned 14 days (T1), one month (T2) and three months (T3) after stent placement. These visits will take 30 minutes. T2 and T3 are extra visits compared to standard of care.

# 1. INTRODUCTION AND RATIONALE

Perihilar cholangiocarcinoma (pCCA) is the most common malignancy of the bile ducts. Unfortunately, most patients are ineligible for curative-intent resection because of metastatic disease, locally advanced (i.e. unresectable) disease, or co-morbidity precluding a major liver resection. In the palliative setting, the median overall survival is only 6-10 months (1-3).

The key to successful palliative treatment is adequate biliary drainage to improve the patient's wellbeing and to allow for palliative systemic therapy. Discussion remains whether palliative biliary drainage is best achieved percutaneously or endoscopically (4-6). Regardless of a percutaneous or an endoscopic approach, biliary drainage for pCCA has a high risk of post-drainage cholangitis and most patients require multiple reinterventions to improve biliary drainage (1, 7). As a result of these complications, 90-day mortality was 35% after initial palliative drainage in 186 patients with (suspected) unresectable pCCA in the Netherlands (1). Notably, the majority of patients with 90-day mortality have no metastatic disease, suggesting that mortality is mainly caused by insufficient biliary drainage and/or drainage-related complications (1). Failure of current management of biliary obstruction in unresectable pCCA is also reflected by the very low percentage (<10%) of patients who receive palliative systemic chemotherapy.

Endoscopic biliary drainage with plastic stents in one or more segments of the liver is the most common technique in the Netherlands (1). The main problem of this approach is that the stents cause bacterial colonization of the previously sterile intrahepatic bile ducts, because the stents cross the ampulla. Cholangitis often develops, especially if undrained segments become colonized. Percutaneous transhepatic biliary drainage is the alternative approach. External biliary drainage can be accomplished without passing the tumor and the ampulla. The drawback of external-only drainage is that patients typically dehydrate and develop metabolic disturbance. The bile fluids can be readministered with a nasoduodenal feeding tube. However, such a tube impacts the quality of life. Moreover, cholangitis can still develop from skin bacteria gaining access to the biliary tree with external biliary drainage. Also, external drainage suffers from a high risk of catheter dislodgement. Therefore, percutaneous biliary catheters are typically internalized: that is, side holes in the intrahepatic part of the catheter collect the bile and deliver the bile via the tip of the catheter which is positioned beyond the ampulla in the duodenum. The downside of an internalized percutaneous biliary catheter is the same as an endoscopic plastic stent: the ampulla is crossed and biliary bacterial colonization occurs. Percutaneously placed catheters are typically flushed with normal saline to avoid clogging of the side holes. However, flushing has the theoretical downside of causing colonized bile to end up in undrained segments and cause cholangitis.

The only method to avoid colonization of the bile ducts is percutaneous placement of uncovered self-expandable metal stents (SEMS) that do not cross the ampulla. After stent placement, the tract is sealed (e.g., with glue) without leaving a catheter.
Only patients with resectable or unresectable perihilar malignancy are eligible for SEMS placement, since it is not possible to endoscopically remove a SEMS and long-term effects of a SEMS for benign disease are unknown. However, a frequent diagnostic challenge is the distinction of benign and malignant biliary duct strictures. Benign diseases, such as Mirizzi Syndrome and IgG4-related sclerosing cholangitis, can mimic perihilar malignancy as well.(11, 12) In many patients with suspected pCCA, histological confirmation of malignant disease is lacking at the time of the first drainage procedure. In these patients, the probability of the stricture being malignant is then estimated based on clinical symptoms by the multidisciplinary hepatobiliary team.

Painless jaundice is the presenting symptom in 90% of pCCA patients. Fifty-six percent of pCCA patients have systemic signs of malignancy (i.e., anorexia, weight loss, and fatigue) at their initial presentation.(8) Jaundice can also be present in advanced gallbladder cancer or intrahepatic cholangiocarcinoma, 30% and 20% at presentation, respectively.(9, 10) When a suspicion of a primary malignant perihilar stricture is seen on imaging, it can be difficult to distinguish these different entities.

Absence of systemic signs of malignancy and fluctuation or spontaneous decrease of a total bilirubin level before start of any treatment can suggest potential benign origin. If a combination of high suspicion of primary malignant perihilar obstruction on imaging and an increasing total bilirubin level is present, according to unpublished data from our own center only a very small number of cases (less than 5%) will eventually turn out to be benign disease. Histological confirmation can be obtained through frozen section procedure before the SEMS is placed, when there is any doubt of malignant disease. During the procedure biopsies and brushes can be performed on default to obtain tissue diagnosis.

We hypothesize that direct SEMS placement in patients with unresectable primary malignant perihilar obstruction minimizes post-drainage cholangitis and mortality, requires fewer reinterventions, and increases the rate of patients receiving palliative chemotherapy.

## 2. OBJECTIVES

Primary Objectives:

- 6-month overall survival (OS).

Secondary Objective(s):

Pilot Cohort and overall analysis

- Stent-related complications according to Clavien-Dindo grading system within 90 days (see Table 2.);
- Absolute and relative (%) bilirubin decrease after 14 days;
- Number of scheduled and unscheduled reinterventions within 90 days.
- Infectious biliary complications (i.e. cholangitis and cholecystitis);
- Technical success of stent placement at initial drainage procedure;
- Bile culture results;
- Proportion of patients that started with palliative chemotherapy;
- Cost-effectiveness.

Expansion cohort

- Quality of Life (QoL).

# 3. STUDY DESIGN

Pilot cohort (10 patients)

In preparation of the design of a phase-II study, we aim to perform a proof-of-concept pilot study at Erasmus Medical Center, Rotterdam, The Netherlands, including 10 patients in order to proof safety and feasibility of direct SEMS placement for palliative treatment of a primary malignant perihilar stricture. The expected inclusion period is one year. After the pilot we will evaluate the safety and feasibility of the method as well as complication rates. Based on our findings we will amend the protocol before continuing with the formal phase-II trial.

Expansion cohort (27 patients)

After the inclusion of the first 10 patients has been completed, an expansion cohort will be added in which UMC Utrecht, Amsterdam UMC and Oslo University Hospital as participating centers will be added, in order to perform a multicenter phase-II trial. Following the analyses of the first 10 patients, another 27 patients will be included to assess the effectiveness and safety of direct SEMS placement for palliative treatment of a primary malignant perihilar stricture. In total 37 patients will be included and analyzed for the study endpoints. The expected inclusion period of the additional 27 patients is two years.

Second expansion cohort (30 patients)

Nine months (July 1$^{st}$, 2022) after approval for the first expansion cohort of 27 patients, we have included 20 additional patients in Erasmus MC. For the first 30 patients (10+20), the outcomes are compared with our historical cohort for endoscopic biliary drainage in Table 1.

*Table 1*

| | Number of patients | Reintervention within 90 days | Mortality within 90 days | Administration of systemic chemotherapy |
|---|---|---|---|---|
| Endoscopic drainage – retrospective cohort | 90 | 75% | 35% | 20% |
| Primary percutaneous stenting - TESLA trial | 30 | 6% | 3% | 75% |

Because of these favorable results, we are currently designing a multicenter randomized controlled trial (RCT) to compare this intervention with the standard of care (i.e. endoscopic biliary drainage). Six additional centers will participate in this trial. To avoid any aspect of

learning curve regarding the intervention within the RCT, each participating center is required to perform the study intervention in 5 patients in this phase II study, before accruing patients in the RCT. Therefore, a second expansion cohort of 30 patients (i.e. 5 patients per center for 6 centers) is required.

A total of 67 patients (10+27+30) will be included and analyzed for the study endpoints. The expected inclusion period of the additional 30 patients, in six participating centers, is one year.

# 4. STUDY POPULATION

## 4.1 Population (base)

Patients with high suspicion of a primary malignant perihilar stricture who cannot undergo a curative-intent resection are eligible for inclusion. Eligibility is established during the multidisciplinary meeting. The definitive diagnosis of pCCA is established based on histopathology from endoscopic or percutaneous ultrasound-guided biopsy or on cytology obtained using endoscopic brush or fine-needle aspiration from the primary tumor or from a metastasis. In absence of histopathological confirmation, the multidisciplinary hepatopancreaticobiliary team form an opinion on the probable diagnosis of pCCA based on clinical symptoms, radiological and endoscopic imaging and laboratory tests(13). Informed consent is obtained at the outpatient clinic.

## 4.2 Inclusion criteria

In order to be eligible to participate in this study, a subject must meet the following criteria:

- Written informed consent must be given according to ICH/GCP, and national/local regulations.
- Unresectable primary malignant perihilar obstruction on imaging with histopathological confirmation or high clinical suspicion (as determined by the multidisciplinary hepatobiliary team)
  And
- Symptomatic hyperbilirubinemia (a combination of a total bilirubin level >20 mmol/l, and/or jaundice and/or loss of appetite and/or dark urine and/or steatorrhea)

## 4.3 Exclusion criteria

A potential subject who meets any of the following criteria will be excluded from participation in this study:

- Fluctuation or spontaneous decrease of a total bilirubin level before start of any treatment suggesting potential benign origin.
- Patients who underwent previous drainage procedures endoscopically or percutaneously with an internalized biliary catheter.
- Clinical signs of cholangitis. Cholangitis was defined as both fever (i.e. body temperature >38.5°C) and leucocytosis (i.e. ≥10 *$10^9$/L) without clinical or radiological evidence of acute cholecystitis (14). Patients who underwent ERCP are eligible, providing no papillotomy was performed or stent was placed and there are no signs of cholangitis.

### 4.4 Sample size calculation

Pilot cohort (10 patients)

A total of 10 patients will be included in this pilot study. This number will be sufficient to investigate treatment feasibility and to identify any bottlenecks in the protocol that should be adjusted for the definitive trial protocol. Taking into account that pCCA is a rare disease, the number of 10 patients will ensure a feasible time frame.

Expansion cohort (27 patients)

To detect a 20% mortality decrease compared with our historical cohort (45% mortality within 6 months) we need to include 37 patients using an alpha of 0.05 and power of 80%. We expect to finish patient accrual within two years.

Following the very promising results in the first 10 patients, another 27 patients will be included to assess the effectiveness and safety of direct SEMS placement for palliative treatment of a primary malignant perihilar stricture.

Taking into account that pCCA is a rare disease, another 27 patients will ensure a feasible time frame for study completion.

Extra expansion cohort (30 patients)

We want all current and future participating centers to perform the intervention in 5 patients in this study before entering the upcoming RCT. For this reason, another 30 patients will be included to assess the effectiveness and safety of direct SEMS placement for palliative treatment of a primary malignant perihilar stricture. The expected inclusion period of the additional 30 patients, in at least four participating centers, is one year.

Total cohort: 10 + 27 + 30 = 67 patients.

# 5. TREATMENT OF SUBJECTS

## 5.1 Investigational product/treatment

Preprocedural intravenous antibiotic prophylaxis is administered (cefuroxime 1500 mg/metronidazole 500 mg; conform institutional ERCP protocol). Procedural sedation and analgesia is performed. An interventional radiologist performs the procedure: (1) ultrasound- and fluoroscopy guided percutaneous transhepatic biliary drainage, (2) bridging significant ductal strictures with a wire and catheter and (3) placement of an uncovered SEMS, (4) without cannulation of the ampulla. Bile cultures are routinely taken. Depending on the tumor size and its localization, right intercostal and/or left epigastric access is chosen. The initial percutaneous transhepatic bile duct puncture is performed with a 21G Chiba needle. Then, a Neffset sheath is placed (Cook Medical) and using a 0.032-0.038 inch glideguidewire with 4 Fr catheter is advanced through the bile duct stricture without passing the ampulla. After crossing the stricture, the glidewire is preferable replaced by a stiff Amplatz guidewire. The Neffset sheath is replaced by a vascular sheath that accommodates the biliary stent (7 or 8Fr). Before stent insertion, the stricture (occlusion) is dilated with a balloon catheter that is pulled over the guidewire (diameter 6 to 8 mm). Then, a biliary stent (HILZO Biliary stent, uncovered straight type, 10mm diameter and 6 or 8cm length) is inserted, still with the wire not passing the ampulla. Bilateral stents are placed when suboptimal drainage of contralateral bile ducts is seen after placement of a single uncovered SEMS. The tract is sealed with Avitene (BD, NJ, USA) mixed with 8 ml of iodine contrast agent without leaving an external drain, unless the SEMS is not sufficiently expanded. When histological confirmation is lacking, during the same procedure percutaneous (forceps) biopsies and brush cytology are taken to confirm the presence of pCCA by frozen section before SEMS-placement, in order to prevent placement of (non-removable) SEMS in patients who have potentially non-malignant disease. Whenever the stricture cannot be passed with a wire, and stent placement is not feasible, an external biliary drain is placed. Crossing of the ampulla is still not performed (no internal-external drainage). A second attempt of recanalization and stenting is performed after 3 or more days.

Intravenous antibiotics (cefuroxime 1500 mg/metronidazole 500 mg) are routinely administered during hospitalization. After discharge oral antibiotics (Amoxicillin/clavulanate 500/125 mg) are administered during five days.
The radiologist reports whether stent placement was technically successful, and whether a second drainage procedure is indicated (e.g., in case the stricture cannot be passed in the first attempt). Primary technical success is defined as successful passage of the stricture and stent placement. Successful drainage is defined as a bilirubin below 50 mmol/l or a

reduction in bilirubin level of at least 50% within 14 days after drainage. Secondary technical success is defined as successful passage of the stricture and stent placement 3 days or more after the initial procedure.

### 5.2 Use of co-intervention

Not applicable.

### 5.3 Escape medication (if applicable)

Not applicable.

# 6. INVESTIGATIONAL PRODUCT

Not applicable, as this study does not involve an investigational product.

## 6.1 Name and description of investigational product(s)

Not applicable, as this study does not involve an investigational product.

## 6.2 Summary of findings from non-clinical studies

Not applicable, as this study does not involve an investigational product.

## 6.3 Summary of findings from clinical studies

Not applicable, as this study does not involve an investigational product.

## 6.4 Summary of known and potential risks and benefits

Not applicable, as this study does not involve an investigational product.

## 6.5 Description and justification of route of administration and dosage

Not applicable, as this study does not involve a medicinal product.

## 6.6 Dosages, dosage modifications and method of administration

Not applicable, as this study does not involve a medicinal product.

## 6.7 Preparation and labelling of Investigational Medicinal Product

Not applicable, as this study does not involve a medicinal product.

## 6.8 Drug accountability

Not applicable, as this study does not involve a medicinal product.

# 7. NON-INVESTIGATIONAL PRODUCT

## 7.1 Name and description of non-investigational product(s)

Patients will undergo percutaneous transhepatic biliary drainage with SEMS placement. Name and description are summarized in the Product Characteristics, see appendix A.

## 7.2 Summary of findings from non-clinical studies

These are summarized in the Product Characteristics, see appendix A.

## 7.3 Summary of findings from clinical studies

These are summarized in the Product Characteristics, see appendix A.

## 7.4 Summary of known and potential risks and benefits

These are summarized in the Product Characteristics, see appendix A.

## 7.5 Description and justification of route of administration and dosage

Not applicable, as this study does not involve a medicinal product.

## 7.6 Dosages, dosage modifications and method of administration

Not applicable, as this study does not involve a medicinal product.

## 7.7 Preparation and labelling of Non Investigational Medicinal Product

Not applicable, as this study does not involve a medicinal product.

## 7.8 Drug accountability

Not applicable, as this study does not involve a medicinal product.

# 8. METHODS

## 8.1 Study parameters/endpoints

### 8.1.1 Main study parameter/endpoint

- OS, defined as the interval between the day of intervention and date of death or last follow-up. This will be estimated using the Kaplan-Meier method;

### 8.1.2 Secondary study parameters/endpoints

- Stent-related complications according to Clavien-Dindo grading system within 90 days (see Table 2.).
- Absolute and relative (%) bilirubin decrease after 14 days.
- Number of scheduled and unscheduled reinterventions within 90 days.
- Infectious biliary complications (i.e. cholangitis and cholecystitis);
- Technical success of stent placement at initial drainage procedure;
- Bile culture results;
- QoL, measured with the EORTC Quality of Life Questionnaire C-30 (QLQ-C30) and its biliary cancer module (QLQ-BIL21) (see Appendix C and D);
- Proportion of patients that became eligible for palliative chemotherapy, based on bilirubin level below 20 mmol/l and good performance status (ECOG < 2);
- Cost-effectiveness.

### 8.1.3 Other study parameters (if applicable)

Not applicable.

## 8.2 Randomisation, blinding and treatment allocation

Not applicable.

## 8.3 Study procedures

**<u>Before stent placement</u>**

- Full eligibility check as described in section 4.
- Written informed consent

**<u>Laboratory tests</u>**

**During stent placement**

- Bile cultures and next-generation sequencing of bile cell-free DNA.
- When histological confirmation is lacking:

- Percutaneous liver- or peritoneal biopsies are performed (only if liver- or distant metastases are present).
  Or
- Intraductal biopsies and brush cytology are performed as follows:
  - Samples from the stricture are taken using a cytology brush by moving the brush five times back and forth through the stricture.
  - Brush is performed two times and both brushes are placed in Cytolyt medium. Cytology and Next Generation Sequencing will be performed (on indication) at the Pathology Department (according to Erasmus MC, UMC Utrecht, Amsterdam UMC and Oslo University Hospital protocol).
  - Three or four intraductal forceps biopsies are performed.
  - Histological confirmation can be obtained through frozen section procedure before the SEMS is placed.

**After stent placement (i.e. follow-up)**

- Serum bilirubin is measured at T1(14 and 28 days). One blood sample of 10 ml will be taken.

**<u>Follow up</u>**

Follow-up appointments are planned as deemed necessary by the treating specialist, but at least 14 days (T1), one month (T2) and three months (T3) after stent placement. Each follow-up will take 30 minutes. During T1, two blood samples (20 ml in total) will be taken. This corresponds to the standard of care. During the other visits blood samples will only be taken by discretion of the treating physician. If patients returned to their referring hospital or if visits are too stressfull, the T2 and T3 visits will be telephone calls. During these calls patients will answer questions about the presence of fever, jaundice and abdominal pain. 1 year after inclusion the treating physician will be contacted about late complications and vital status. Complications according to the Clavien-Dindo grading system (see Table 2.) and reinterventions are scored at each follow-up.

**<u>Quality of life</u>**

Patients complete questionnaires (QLQC30/BIL21) (see Appendix C and D) at the day of informed consent, and at 14 days, 28 days, and 90 days after the first intervention.

**Table 2. Classification of interventional complications**

| | |
|---|---|
| Grade I | Any deviation from the normal postoperative course without the need for pharmacological treatment or surgical, endoscopic, and radiological interventions<br>Allowed therapeutic regimens are: drugs as antiemetics, antipyretics, analgetics, diuretics, electrolytes, and physiotherapy. This grade also includes wound infections opened at the bedside |
| Grade II | Requiring pharmacological treatment with drugs other than such allowed for grade I complications |
| Grade III | Requiring surgical, endoscopic or radiological intervention |
| Grade IIIa | Intervention not under general anaesthesia |
| Grade IIIb | Intervention under general anaesthesia |
| Grade IV | Life-threatening complication (including CNS complications)* requiring IC/ICU management |
| Grade IVa | Single organ dysfunction (including dialysis) |
| Grade IVb | Multiorgan dysfunction |
| Grade V | Death of a patient |

*Brain hemorrhage, ischemic stroke, subarachnoidal bleeding, but excluding transient ischemic attacks. CNS, central nervous system; IC, intermediate care; ICU, intensive care unit.

## 8.4 Withdrawal of individual subjects

Subjects can leave the study at any time for any reason if they wish to do so without any consequences. The investigator can decide to withdraw a subject from the study for urgent medical reasons.

### 8.4.1 Specific criteria for withdrawal (if applicable)

Not applicable.

## 8.5 Replacement of individual subjects after withdrawal

Not applicable, no replacement will take place.

## 8.6 Follow-up of subjects withdrawn from treatment

If patients returned to their referring hospital or if visits are burdensome, the T2 and T3 visits will be telephone calls. During these calls patients will answer questions about the presence of fever, jaundice and abdominal pain.

## 8.7 Premature termination of the study

The Sponsor may decide to terminate the study prematurely based on the following criteria:

- There is evidence of an unacceptable risk for study patients; The safety and feasibility will be monitored after 5 patients. The presence of sepsis,

cholangitis, bleeding, number of scheduled and unscheduled reinterventions and mortality rate within 90 days will be evaluated.

- There is reason to conclude that it will not be possible to collect the data necessary to reach the study objectives and it is therefore not ethical to continue enrolment of more patients; for example insufficient enrolment that cannot be improved.

The Sponsor will promptly notify all concerned investigators, the Ethics Committee(s) and the regulatory authorities of the decision to terminate the study. The Sponsor will provide information regarding the time lines of study termination and instructions regarding treatment and data collection of enrolled patients.

# 9. SAFETY REPORTING

## 9.1 Temporary halt for reasons of subject safety

In accordance to section 10, subsection 4, of the WMO, the sponsor will suspend the study if there is sufficient ground that continuation of the study will jeopardise subject health or safety. The sponsor will notify the accredited METC without undue delay of a temporary halt including the reason for such an action. The study will be suspended pending a further positive decision by the accredited METC. The investigator will take care that all subjects are kept informed.

## 9.2 AEs, SAEs and SUSARs

### 9.2.1 Adverse events (AEs)

Adverse events are defined as any undesirable experience occurring to a subject during the study, whether or not considered related to the experimental intervention. Adverse events (grade 3 or higher) reported spontaneously by the subject or observed by the investigator or his staff will be recorded.

### 9.2.2 Serious adverse events (SAEs)

A serious adverse event is any untoward medical occurrence or effect that

- results in death;
- is life threatening (at the time of the event);
- requires hospitalization or prolongation of existing inpatients' hospitalization;
- results in persistent or significant disability or incapacity;
- is a congenital anomaly or birth defect; or
- any other important medical event that did not result in any of the outcomes listed above due to medical or surgical intervention but could have been based upon appropriate judgement by the investigator.

An elective hospital admission will not be considered as a serious adverse event.

Progression of the disease under study is not considered an SAE unless it meets any of the seriousness criteria mentioned above.

The principal investigator will decide whether or not the SAE is related to study treatment. The decision will be recorded on the serious adverse event report. The assessment of causality is made by the local investigator using the following:

| RELATIONSHIP | DESCRIPTION |
|---|---|
| UNRELATED | There is no evidence of any causal relationship |
| RELATED | There is evidence to suggest a causal relationship |

The sponsor will report the SAEs through the web portal *ToetsingOnline* to the accredited METC that approved the protocol, within 7 days of first knowledge for SAEs that result in death or are life threatening followed by a period of maximum of 8 days to complete the initial preliminary report. All other SAEs will be reported within a period of maximum 15 days after the sponsor has first knowledge of the serious adverse events.

#### 9.2.3 Suspected unexpected serious adverse reactions (SUSARs)

Not applicable.

### 9.3 Annual safety report

Not applicable.

### 9.4 Follow-up of adverse events

All AEs will be followed until they have abated, or until a stable situation has been reached. Depending on the event, follow up may require additional tests or medical procedures as indicated, and/or referral to the general physician or a medical specialist.
SAEs need to be reported till end of study within the Netherlands, as defined in the protocol.

### 9.5 Data Safety Monitoring Board (DSMB)

A Data and Safety Monitoring Board will not be installed. Monitoring of data and safety will be performed as described in the monitoring plan (see chapter 12.2: Monitoring and Quality Assurance).

# 10. STATISTICAL ANALYSIS

## 10.1 Primary study parameter(s)

Pilot cohort and overall analysis

The primary outcomes for safety are OS is defined as the interval between the day of intervention and date of death or last follow-up. This will be estimated using the Kaplan-Meier method.

## 10.2 Secondary study parameter(s)

Pilot cohort and overall analysis

The secondary outcomes for safety and feasibility are the stent-related complications, the amount of reïnterventions in 90 days, absolute and relative (%) decrease of bilirubin in 14 days, infectious biliary complications (i.e. cholangitis and cholecystitis), technical success of stent placement at initial drainage procedure, proportion of patients that started with palliative chemotherapy and cost-effectiveness. The proportion and 95% confidence interval will be calculated for the continuous variables. The median and interquartile range will be measured for the numeric variables.

Expansion cohort

QoL is measured with (QLQC30/BIL21) (see Appendix C and D). Repeated measurement analysis will be used to evaluate within group differences. the repeated measurements will be analyzed separately using linear mixed models with correction for the baseline score (added as covariate). The single items in the QLQ-C30 will be analyzed using (ordinal) logistic regression with random effects. The BIL21 questionnaire will also be used as utility measure for the cost-effectiveness analysis.

We will perform a cost-effectiveness analysis to evaluate the impact of percutaneous placement of metal stents in patients with unresectable pCCA. The cost-effectiveness analysis from the healthcare sector perspective will consider direct medical costs of both strategies. Prospective patient-level cost data collection for each patient in the trial would be ideal but is too expensive to collect. Instead, we will model costs in a decision model using probabilities of events and unit costs of interventions.

Data from a comparable matched cohort of both participating centers of patients treated with endoscopic drainage in the period before the current trial, will be used as a comparison. The health effects will be expressed in quality-adjusted life years (QALYs). The QALY combines the number of life years with the quality of life measured with the EQ-5D utilities. The cost-effectiveness of percutaneous placement of metal stents will be expressed as the incremental costs per QALY gained to allow comparison with other (unrelated) health care

interventions. In addition, we will determine the degree of uncertainty regarding the cost-effectiveness of percutaneous placement of metal stents by performing probabilistic sensitivity analyses. We will present the uncertainty of cost-effectiveness estimates using scatter-plots on a cost-effectiveness plane, cost-effectiveness acceptability curves, and value of information analysis. The project leader (Bas Groot Koerkamp) has a PhD in cost-effectiveness analysis.

### 10.3 Other study parameters

Not applicable.

### 10.4 Interim analysis (if applicable)

Interim analyses will not be performed during this pilot study.

# 11. ETHICAL CONSIDERATIONS

## 11.1 Regulation statement

The investigator will ensure that this study is conducted to the standards of Good Clinical Practice, in full conformance with the “Declaration of Helsinki” (64yh World Medical Association General Assembly, latest amendment Fortaleza, Brazil, October 2013), the Dutch laws and regulations with the WMO (“Wet Medisch-wetenschappelijk Onderzoek met mensen”) in particular.

## 11.2 Recruitment and consent

Patients will be asked to participate in this study when they are seen at the outpatient clinic. Initially, the patient will be informed of the nature of the study and will be given pertinent information as to the intended purpose of the study by the treating physician or study coordinator. The procedures and possible hazards to which the patient will be exposed will be explained. The patient will also receive written information (patient information form). After receiving study information, patients will be given enough time to consider whether they wish to participate or not. Prior to the screening evaluation, an informed consent statement, as approved by the Medical Ethical Committee Erasmus MC Rotterdam will be read and signed by the patient, and the responsible physician/coordinating investigator. The patient will be provided with a second original form of the signed informed consent statement. The patient may withdraw from the study at any time.

The investigator shall provide a copy of the information sheet and the signed consent form to the patient and the signed original shall be maintained in the Investigator Site File.

## 11.3 Objection by minors or incapacitated subjects (if applicable)

Not applicable, as no minors or incapacitated subjects are eligible for this study.

## 11.4 Benefits and risks assessment, group relatedness

For eligible patients, curative surgical treatment is not possible and the median survival time is short. Palliative treatment is aimed at obtaining adequate biliary drainage followed by palliative systemic chemotherapy.

90-day mortality with the current standard of care of endoscopic drainage was 35%. The percutaneous intervention is an alternative approach. We hypothesize that direct percutaneous SEMS placement in patients with unresectable pCCA reduces post-drainage cholangitis and mortality, requires fewer reinterventions, and increases the rate of patients receiving palliative chemotherapy. Specific complications related to the percutaneous approach are bleeding and bile leakage, which are uncommon (<5%).

In this study, patients will undergo an invasive procedure with hospital discharge after a few days. This corresponds to the standard of care. Follow-up appointments are planned 14 days (T1), one month (T2) and three months (T3) after stent placement. These visits will take 30 minutes. T2 and T3 are extra visits compared to standard of care.

### 11.5 Compensation for injury

The sponsor/investigator has a liability insurance which is in accordance with article 7 of the WMO.

The sponsor (also) has an insurance which is in accordance with the legal requirements in the Netherlands (Article 7 WMO). This insurance provides cover for damage to research subjects through injury or death caused by the study.
The insurance applies to the damage that becomes apparent during the study or within 4 years after the end of the study.

### 11.6 Incentives (if applicable)

Not applicable, as there is no subsidising party for this study.

# 12. ADMINISTRATIVE ASPECTS, MONITORING AND PUBLICATION

## 12.1 Handling and storage of data and documents

**Patient confidentiality**

Each patient is assigned a unique patient study number at enrolment, which is used to code ( 001, 002 etc.) the patient's identity in the study documents.
The investigator will keep a subject enrolment and identification log that contains the key to the code, i.e. a record of the personal identification data linked to each patient study number. This record is filed at the investigational site and should only be accessed by the investigator and the supporting site staff, and by representatives of the Sponsor or a regulatory agency for the purpose of monitoring visits or audits and inspections.

**Case Report Forms**

Data will be collected on electronic Case Report Forms (CRF) in OpenClinica to document eligibility, safety and efficacy parameters, compliance to treatment schedules and parameters necessary to evaluate the study endpoints. Data to be collected on the e-CRF are derived from the protocol.

**Filing of essential documents**

Essential Documents are those documents that permit evaluation of the conduct of a trial and the quality of the data produced. The essential documents may be subject to, and should be available for, audit by the Sponsor's auditor and inspection by the regulatory authority(/-ies).
The investigator should file all essential documents relevant to the conduct of the study on site in the Investigator Site File. The Sponsor will file all essential documents relevant to the overall conduct of the trial in the Trial Master File. Essential documents should be filed in such a manner that they are protected from accidental loss and can be easily retrieved for review.

**Record retention**

Essential documents should be retained for 15 years after the end of the study (i.e. from date of last patient visit for this study). They should be destroyed after this time.
Source documents (i.e. medical records) of patients should be retained for at least 15 years after the end of the study. Record retention and destruction after this time is subject to the site's guidelines regarding medical records.

### 12.2 Monitoring and Quality Assurance

Based on the guideline by the NFU (Dutch Federation of University Medical Centers) about quality insurance in human research (“Kwaliteitsborging van mensgebonden onderzoek”) we qualify the risk of this study as ‘low’.

On behalf of the Sponsor the monitor will perform on-site monitoring visits to verify that the rights and well-being of patients are protected, the reported trial data are accurate, complete, and verifiable from source documents and the conduct of the trial is in compliance with the currently approved protocol/amendments(s), with GCP, and with the applicable regulatory requirement(s). Our sourcedata is the electronical health record at Erasmus MC (HiX). If patients returned to their referring hospital or if visits are too stressfull, the T2 and T3 visits will be telephone calls.

A monitor will be appointed prior to the start of the study. The monitor will evaluate at least the safety and outcome parameters at regular intervals, in compliance with the risk class assessment (see monitoring plan and associated risk class A/B/C appendix).

### 12.3 Amendments

Amendments are changes made to the research after a favourable opinion by the accredited METC has been given. All amendments will be notified to the METC that gave a favourable opinion.

### 12.4 Annual progress report

The sponsor/investigator will submit a summary of the progress of the trial to the accredited METC once a year. Information will be provided on the date of inclusion of the first subject, numbers of subjects included and numbers of subjects that have completed the trial, serious adverse events/ serious adverse reactions, other problems, and amendments.

### 12.5 Temporary halt and (prematurely) end of study report

The investigator/sponsor will notify the accredited METC of the end of the study within a period of 8 weeks. The end of the study is defined as the last patient’s last visit.

The sponsor will notify the METC immediately of a temporary halt of the study, including the reason of such an action.

In case the study is ended prematurely, the sponsor will notify the accredited METC within 15 days, including the reasons for the premature termination.

Within one year after the end of the study, the investigator/sponsor will submit a final study

report with the results of the study, including any publications/abstracts of the study, to the accredited METC.

## 12.6 Public disclosure and publication policy

Publications resulting from this study will be submitted to peer-reviewed journals. The projectleader and co-investigators will prepare the manuscript together with those who substantially contributed to the study. Specification for authorship have been summarized in the appendix (appendix B). Any publication, abstract or preservation based on patients included in this study must be approved by the projectleader and the co-investigators. This is applicable to any individual patient registered in the trial, or any subgroup of the trial patients. Such a publication cannot include any comparisons between randomized treatment arms or an analysis of any of the study end-points unless the final results of the trial have already been published. Information about this study is also included in an online overview of medical research studies, www.trialregister.nl.

# 13. STRUCTURED RISK ANALYSIS

## 13.1 Potential issues of concern

Skipped.

## 13.2 Synthesis

Section 13.1 is skipped, because the intervention will be done with a registered product which is to be used within the indication. The risks of transhepatic biliary drainage include cholangitis (8%), pancreatitis (3%), bleeding which is limited to liver and bile ducts or bleeding in the abdominal cavity (2%), and leak of the bile into the abdominal cavity or into the space around the lung (<1%) (15). With a percutaneous approach without crossing the ampulla the risk of post-drainage cholangitis and mortality is expected to be reduced

# 14. REFERENCES

1. Gaspersz MP, van Vugt JLA, Roos E, Coelen RJS, Vugts J, M.J. B, et al. Success, Complication, and Mortality Rate of Initial Biliary Drainage in Patients with Unresectable Perihilar Cholangiocarcinoma. unpublished. 2018.
2. Jarnagin WR, Fong Y, DeMatteo RP, Gonen M, Burke EC, Bodniewicz BJ, et al. Staging, resectability, and outcome in 225 patients with hilar cholangiocarcinoma. Ann Surg. 2001;234(4):507-17; discussion 17-9.
3. Chaiteerakij R, Harmsen WS, Marrero CR, Aboelsoud MM, Ndzengue A, Kaiya J, et al. A new clinically based staging system for perihilar cholangiocarcinoma. Am J Gastroenterol. 2014;109(12):1881-90.
4. Wiggers JK, Groot Koerkamp B, Coelen RJ, Doussot A, van Dieren S, Rauws EA, et al. Percutaneous Preoperative Biliary Drainage for Resectable Perihilar Cholangiocarcinoma: No Association with Survival and No Increase in Seeding Metastases. Ann Surg Oncol. 2015;22 Suppl 3:S1156-63.
5. Moole H, Dharmapuri S, Duvvuri A, Dharmapuri S, Boddireddy R, Moole V, et al. Endoscopic versus Percutaneous Biliary Drainage in Palliation of Advanced Malignant Hilar Obstruction: A Meta-Analysis and Systematic Review. Can J Gastroenterol Hepatol. 2016;2016:4726078.
6. Paik WH, Park YS, Hwang JH, Lee SH, Yoon CJ, Kang SG, et al. Palliative treatment with self-expandable metallic stents in patients with advanced type III or IV hilar cholangiocarcinoma: a percutaneous versus endoscopic approach. Gastrointest Endosc. 2009;69(1):55-62.
7. Sangchan A, Kongkasame W, Pugkhem A, Jenwitheesuk K, Mairiang P. Efficacy of metal and plastic stents in unresectable complex hilar cholangiocarcinoma: a randomized controlled trial. Gastrointest Endosc. 2012;76(1):93-9.
8. Blechacz B. Cholangiocarcinoma: Current Knowledge and New Developments. Gut Liver. 2017;11(1):13-26.
9. Alvaro D, Bragazzi MC, Benedetti A, Fabris L, Fava G, Invernizzi P, et al. Cholangiocarcinoma in Italy: A national survey on clinical characteristics, diagnostic modalities and treatment. Results from the "Cholangiocarcinoma" committee of the Italian Association for the Study of Liver disease. Dig Liver Dis. 2011;43(1):60-5.
10. Hawkins WG, DeMatteo RP, Jarnagin WR, Ben-Porat L, Blumgart LH, Fong Y. Jaundice predicts advanced disease and early mortality in patients with gallbladder cancer. Ann Surg Oncol. 2004;11(3):310-5.
11. Clemente G, Tringali A, De Rose AM, Panettieri E, Murazio M, Nuzzo G, et al. Mirizzi Syndrome: Diagnosis and Management of a Challenging Biliary Disease. Can J Gastroenterol Hepatol. 2018;2018:6962090.
12. Corvera CU, Blumgart LH, Darvishian F, Klimstra DS, DeMatteo R, Fong Y, et al. Clinical and pathologic features of proximal biliary strictures masquerading as hilar cholangiocarcinoma. J Am Coll Surg. 2005;201(6):862-9.
13. Coelen RJS, Gaspersz MP, Labeur TA, van Vugt JLA, van Dieren S, Willemssen F, et al. Validation of the Mayo Clinic Staging System in Determining Prognoses of Patients With Perihilar Cholangiocarcinoma. Clin Gastroenterol Hepatol. 2017;15(12):1930-9 e3.
14. Wiggers JK, Coelen RJ, Rauws EA, van Delden OM, van Eijck CH, de Jonge J, et al. Preoperative endoscopic versus percutaneous transhepatic biliary drainage in potentially resectable perihilar cholangiocarcinoma (DRAINAGE trial): design and rationale of a randomized controlled trial. BMC Gastroenterol. 2015;15:20.
15. Burke DR, Lewis CA, Cardella JF, Citron SJ, Drooz AT, Haskal ZJ, et al. Quality improvement guidelines for percutaneous transhepatic cholangiography and biliary drainage. J Vasc Interv Radiol. 2003;14(9 Pt 2):S243-6.

## APPENDICES

### Appendix A: Product characteristics SEMS

At Erasmus MC two different uncovered stents are used to prevent there are no stents available if there is a short of stack at one of the manufacturers. There are no differences in length or diameter.

*The product characteristics of the ‘ HILZO Biliary Uncovered Stent, BCM ’ is available via the link displayed below on page 5.*

*https://www.kebomed.nl/files/169/hilzo_stents_catalogue.pdf*

*The product characteristics of the ‘WALLSTENT-UNI™ Endoprosthesis Self-Expanding Stent’ is available via the link displayed below.*

http://genodynamic.ro/wp-content/uploads/2015/03/DINPER2042EF_Wallstent_Brochure_English.pdf

## Appendix B: Richtlijn auteurschappen

**Algemene overwegingen:**

De meeste tijdschriften maken een onderscheid tussen:

a) *hoofdauteurs boven artikel:*

deze staan vermeld boven het artikel. Dit aantal is vaak gelimiteerd tot bijvoorbeeld 10 personen ("The British Journal of Surgery holds the view that in the context of surgical publishing most articles are unlikely to involve significant contributions from more than ten authors").

b) *medeonderzoekers = 'collaborators' = leden van de onderzoeksgroep onder artikel:*

alle leden van de onderzoeksgroep staan vermeld aan het einde van het artikel als 'collaborators'. Deze namen worden allen vermeld in PubMed. De bijdrage van de collaborators die niet boven het artikel vermeld staan verschilt inhoudelijk van de bijdrage van hen die wel boven het artikel vermeld staan. Ook deze medeonderzoekers moeten hun positie echter 'verdienen'. Het baart de Editorial Board van bijvoorbeeld Br J Surg zorgen dat in het decembernummer van 2014 een artikel is verschenen waarin 98 patiënten worden beschreven door 54 collaborators. Naar de mening van de editors moet de positie van iedere collaborator verdedigbaar en gefundeerd zijn volgens de richtlijnen van de International Committee of Medical Journal Editors (www.icmje.org). Vrijwel alle internationale tijdschriften committeren zich aan deze richtlijnen:

"The ICMJE recommends that authorship be based on the following 4 criteria:

• Substantial contributions to the conception or design of the work; or the acquisition, analysis, or interpretation of data for the work; AND
• Drafting the work or revising it critically for important intellectual content; AND
• Final approval of the version to be published; AND
• Agreement to be accountable for all aspects of the work in ensuring that questions related to the accuracy or integrity of any part of the work are appropriately investigated and resolved."

"All those designated as authors should meet all four criteria for authorship, and all who meet the four criteria should be identified as authors. Those who do not meet all four criteria should be acknowledged. These authorship criteria are intended to preserve the status of authorship for those who deserve credit and can take responsibility for the work. The criteria are not intended for use as a means to disqualify colleagues from authorship who otherwise meet authorship criteria by denying them the opportunity to meet criterion #1, 2 or 3. Therefore, all

individuals who meet the first criterion should have the opportunity to participate in the review, drafting, and final approval of the manuscript.”

c) *personen in acknowledgements:*

personen die een bijdrage hebben geleverd aan de totstandkoming van het artikel, maar die niet kwalificeren als medeonderzoekers kunnen vermeld worden aan het einde van het artikel onder ‘acknowledgements’. Dit kunnen datamanagers zijn, maar bijvoorbeeld ook geïnterviewde collegae. In de Erasmus MC ‘guidelines on authorship’ staat over dit onderwerp o.a. het volgende vermeld: “A co-authorship is not justified by the routine provision of data or material, or by ensuring the necessary funding. Sufficient acknowledgement of such contributions can be provided by a mention in the ‘acknowledgements’ or in an overview of those who have contributed.”

## Appendix C: EORTC QLQ-C30, version 3.0 (Dutch version)

Wij zijn geïnteresseerd in bepaalde dingen over u en uw gezondheid. Wilt u alle vragen zelf beantwoorden door het getal te omcirkelen dat het meest op u van toepassing is. Er zijn geen "juiste" of "onjuiste" antwoorden. De informatie die u geeft zal strikt vertrouwelijk worden behandeld.

Patient subject number: |___|___|___|
Uw geboortedatum (Dag, Maand, Jaar): …………..
De datum van vandaag (Dag, Maand, Jaar): …………..

| | Helemaal niet | Een beetje | Nogal | Heel erg |
|---|---|---|---|---|
| 1. Heeft u moeite met het doen van inspannende a c tiviteiten zoals het dragen van zware boodschappentas of een koffer? | 1 | 2 | 3 | 4 |
| 2. Heeft u moeite met het maken van een lange wandeling? | 1 | 2 | 3 | 4 |
| 3. Heeft u moeite met het maken van een korte wandeling buitenshuis? | 1 | 2 | 3 | 4 |
| 4.. Moet u overdag in bed of op een stoel blijven? | 1 | 2 | 3 | 4 |
| 5. Heeft u hulp nodig met eten, aankleden, uzelf wassen of naar het toilet gaan? | 1 | 2 | 3 | 4 |

| **Gedurende de afgelopen week:** | **Helemaal niet** | **Een beetje** | **Nogal** | **Heel erg** |
|---|---|---|---|---|
| 6. Was u beperkt bij het doen van uw werk of andere dagelijkse bezigheden? | 1 | 2 | 3 | 4 |
| 7. Was u beperkt in het uitoefenen van uw hobby's of bij andere bezigheden die u in uw vrije tijd doet? | 1 | 2 | 3 | 4 |
| 8. Was u kortademig? | 1 | 2 | 3 | 4 |
| 9. Heeft u pijn gehad? | 1 | 2 | 3 | 4 |
| 10. Had u behoefte om te rusten? | 1 | 2 | 3 | 4 |
| 11. Heeft u moeite met slapen gehad? | 1 | 2 | 3 | 4 |
| 12. Heeft u zich slap gevoeld? | 1 | 2 | 3 | 4 |
| 13. Heeft u gebrek aan eetlust gehad? | 1 | 2 | 3 | 4 |
| 14. Heeft u zich misselijk gevoeld? | 1 | 2 | 3 | 4 |

Wilt u a.u.b. naar de volgende bladzijde gaan

| Gedurende de afgelopen week: | Helemaal niet | Een beetje | Nogal | Heel erg |
|---|---|---|---|---|
| 15. Heeft u overgegeven? | 1 | 2 | 3 | 4 |
| 16. Had u last van obstipatie? (was u verstopt?) | 1 | 2 | 3 | 4 |
| 17. Had u diarree? | 1 | 2 | 3 | 4 |
| 18. Was u moe? | 1 | 2 | 3 | 4 |
| 19. Heeft pijn u gehinderd in uw dagelijkse bezigheden? | 1 | 2 | 3 | 4 |
| 20. Heeft u moeite gehad met het concentreren op dingen, zoals een krant lezen of televisie kijken? | 1 | 2 | 3 | 4 |
| 21. Voelde u zich gespannen? | 1 | 2 | 3 | 4 |
| 22. Maakte u zich zorgen? | 1 | 2 | 3 | 4 |
| 23. Voelde u zich prikkelbaar? | 1 | 2 | 3 | 4 |
| 24. Voelde u zich neerslachtig? | 1 | 2 | 3 | 4 |
| 25. Heeft u moeite gehad met het herinneren van dingen? | 1 | 2 | 3 | 4 |
| 26. Heeft uw lichamelijke toestand of medische behandeling uw familieleven in de weg gestaan? | 1 | 2 | 3 | 4 |
| 27. Heeft uw lichamelijke toestand of medische behandeling u belemmerd in uw sociale bezigheden? | 1 | 2 | 3 | 4 |
| 28. Heeft uw lichamelijke toestand of medische behandeling financiële moeilijkheden met zich meebracht? | 1 | 2 | 3 | 4 |

**Wilt u voor de volgende vragen het getal tussen 1 en 7 omcirkelen dat het meest op u van toepassing is**

29. Hoe zou u uw algehele gezondheid gedurende de afgelopen week beoordelen?

| 1 | 2 | 3 | 4 | 5 | 6 | 7 |
|---|---|---|---|---|---|---|
| Erg slecht | | | | | | Uitstekend |

30. Hoe zou u uw algehele "kwaliteit van het leven" gedurende de afgelopen week beoordelen?

| 1 | 2 | 3 | 4 | 5 | 6 | 7 |
|---|---|---|---|---|---|---|
| Erg slecht | | | | | | Uitstekend |

## Appendix D: EORTC QLQ-BIL21 (Dutch version)

DUTCH

# EORTC QLQ – BIL21

Soms zeggen patiënten dat ze volgende klachten of problemen hebben. Wilt u aangeven in welke mate u deze symptomen of problemen gedurende de afgelopen week heeft ervaren? Wilt u uw antwoord geven door het cijfer te omcirkelen dat het meest op u van toepassing is?

| Gedurende de afgelopen week: | | Helemaal niet | Een beetje | Nogal | Heel erg |
|---|---|---|---|---|---|
| 31. | Heeft u problemen met eten gehad? | 1 | 2 | 3 | 4 |
| 32. | Heeft u het gevoel gehad te snel voldaan te zijn nadat u bent begonnen te eten? | 1 | 2 | 3 | 4 |
| 33. | Heeft u problemen gehad met uw smaakzin? | 1 | 2 | 3 | 4 |
| 34. | Bent u beperkt geweest in het soort voedsel dat u kon eten ten gevolge van uw ziekte of behandeling? | 1 | 2 | 3 | 4 |
| 35. | Zijn uw huid of ogen geel (geelzucht) geweest? | 1 | 2 | 3 | 4 |
| 36. | Heeft u jeuk gehad? | 1 | 2 | 3 | 4 |
| 37. | Heeft u zich zorgen gemaakt omdat uw huid geel was? | 1 | 2 | 3 | 4 |
| 38. | Bent u minder actief geweest dan u had willen zijn? | 1 | 2 | 3 | 4 |
| 39. | Heeft u zich 'futloos' gevoeld? | 1 | 2 | 3 | 4 |
| 40. | Heeft u een tekort aan energie gehad? | 1 | 2 | 3 | 4 |
| 41. | Heeft u 's nachts pijn gehad? | 1 | 2 | 3 | 4 |
| 42. | Heeft u pijn gehad in de maagstreek? | 1 | 2 | 3 | 4 |
| 43. | Heeft u pijn in uw rug gehad? | 1 | 2 | 3 | 4 |
| 44. | Heeft u een opgeblazen gevoel in uw buik gehad? | 1 | 2 | 3 | 4 |
| 45. | Heeft u zich gestrest gevoeld? | 1 | 2 | 3 | 4 |
| 46. | Heeft u het minder makkelijk gevonden om u te amuseren? | 1 | 2 | 3 | 4 |
| 47. | Heeft u zich zorgen gemaakt over uw toekomstige gezondheidstoestand? | 1 | 2 | 3 | 4 |
| 48. | Heeft u zich zorgen gemaakt over uw familie in de toekomst? | 1 | 2 | 3 | 4 |
| 49. | In hoeverre heeft u last gehad van bijwerkingen van uw behandeling? | 1 | 2 | 3 | 4 |
| 50. | Heeft u problemen gehad met drainage slangen/opvangzakken? | 1 | 2 | 3 | 4 |
| 51. | Heeft u zich zorgen gemaakt over gewichtsverlies? | 1 | 2 | 3 | 4 |

© Copyright 2011 EORTC Quality of Life Group. Alle rechten voorbehouden.
