# Supplementary material for: Primary percutaneous stenting for palliative biliary drainage of patients with malignant hilar biliary obstruction: TESLA trial
Source: JHEP Rep. 2025 Sep 11;7(11):101541. doi: 10.1016/j.jhepr.2025.101541 (PMC12529366; doi:10.1016/j.jhepr.2025.101541)
Supplement: Multimedia component 5 [file mmc5.pdf]

JHEP|Reports

Primary percutaneous stenting for palliative biliary drainage of patients with malignant hilar biliary obstruction: TESLA trial

Authors

Stijn Franssen, Merve Rousian, Victorien van Verschuer, …, Diederik Bijdevaate, Adriaan Moelker, Bas Groot Koerkamp

Correspondence

b.grootkoerkamp@erasmusmc.nl (B. Groot Koerkamp).

Graphical abstract

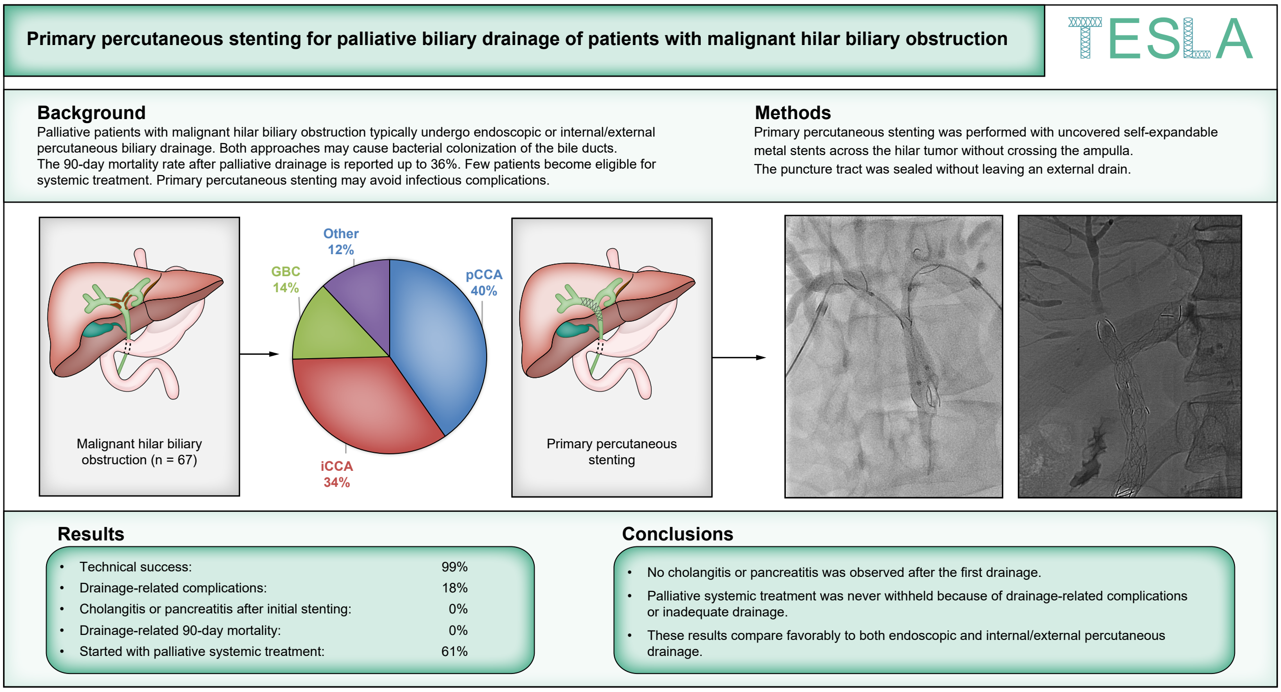

Highlights:

- Primary percutaneous stenting in malignant hilar biliary obstruction (n = 67) was associated with a low complication rate (18.0%).
- Sixty-six patients (98.5%) were technically successfully drained after the first drainage procedure.
- Cholangitis or pancreatitis was never observed after initial stenting.
- No drainage-related 90-day mortality was observed and most patients (61.2%) were started on palliative systemic treatment.

Impact and implications:

This study demonstrates that primary percutaneous stenting in patients with malignant hilar biliary obstruction results in a low rate of drainage-related complications and enables initiation of systemic therapy in the majority of patients. These findings are clinically relevant for gastroenterologists, interventional radiologists, and oncologists aiming to optimize palliative care while minimizing infectious risks. The approach may offer a safe and effective alternative to conventional drainage strategies, although confirmation in comparative trials is needed to support broader implementation.

https://doi.org/10.1016/j.jhepr.2025.101541
© 2025 The Author(s). Published by Elsevier B.V. on behalf of European Association for the Study of the Liver (EASL). This is an open access article under the CC BY license (http://creativecommons.org/licenses/by/4.0/).

Primary percutaneous stenting for palliative biliary drainage of patients with malignant hilar biliary obstruction: TESLA trial[☆]

Stijn Franssen[1], Merve Rousian[1,†], Victorien van Verschuer[1,†], Marco Bruno[2], Michail Doukas[3], Lydi van Driel[2], Marjolein Homs[4], Behnam Mohseny[5], Roeland de Wilde[1], Jeroen de Jonge[1], Wojciech Polak[1], Robert Porte[1], Diederik Bijdevaate[5], Adriaan Moelker[5,‡], Bas Groot Koerkamp[1,*]

JHEP Reports **2025**. vol. 7 | 1–8

Check for updates

Background & Aims: Palliative patients with malignant hilar biliary obstruction typically undergo endoscopic or internal/external percutaneous biliary drainage. Both approaches may cause bacterial colonization of the bile ducts, requiring multiple reinterventions. The 90-day mortality rate after palliative drainage is reported to be up to 36%. Few patients become eligible for systemic treatment. Primary percutaneous stenting may avoid infectious complications. The aim of this study was to investigate primary percutaneous stenting in palliative patients with malignant hilar biliary obstruction.

Methods: We performed a single-arm phase II trial. Primary percutaneous stenting was performed with uncovered self-expandable metal stents across the hilar tumor without crossing the ampulla. The puncture tract was sealed without leaving an external drain. Outcomes included drainage-related severe complications and the proportion of patients receiving systemic treatment after drainage.

Results: From October 2020 until June 2023, 67 patients were included, with perihilar cholangiocarcinoma in 27 patients (40.3%), intrahepatic cholangiocarcinoma in 23 patients (34.3%), gallbladder cancer in nine patients (13.4%), and other tumors in eight patients (12.0%). Drainage-related severe complications within 90 days were observed in 12 patients (17.9%); two patients (3.0%) developed acute cholecystitis, one patient (1.5%) had a biliary leak, three patients (4.5%) had hemorrhage, and six patients (9.0%) had persistent jaundice. No drainage-related 90-day mortality was observed. Cholangitis or pancreatitis was never observed after the first drainage. Palliative systemic treatment was started in 42 patients (62.7%).

Conclusions: Primary percutaneous stenting for patients with malignant hilar biliary obstruction had a low incidence of drainage-related complications without any cholangitis or pancreatitis after the first drainage. Palliative systemic treatment was never withheld because of drainage-related complications or inadequate drainage. These results compare favorably to both endoscopic and internal/external percutaneous drainage.

© 2025 The Author(s). Published by Elsevier B.V. on behalf of European Association for the Study of the Liver (EASL). This is an open access article under the CC BY license (http://creativecommons.org/licenses/by/4.0/).

Introduction

Malignant hilar biliary obstruction (MHBO) is most commonly caused by perihilar cholangiocarcinoma (pCCA) but can also be caused by intrahepatic cholangiocarcinoma (iCCA), gallbladder cancer (GBC), or any other tumor metastasizing to the liver hilum.[1,2] Patients presenting with MHBO are mostly ineligible for surgical resection owing to distant metastases, locally advanced (*i.e.* unresectable) disease, or poor performance status. In the palliative setting of MHBO, the median overall survival (OS) is only 5 months.[3,4]

Patients with MHBO typically present with painless jaundice. The key to successful palliative care is adequate biliary drainage to improve the patient's general well-being and allow for palliative systemic treatment.[5] Endoscopic biliary drainage (EBD) with plastic stents or self-expandable metal stents (SEMS) is the standard of care for MHBO in most guidelines (Fig. 1A).[6,7] The main drawback of the endoscopic approach is bacterial contamination of the intrahepatic bile ducts, caused by the transpapillary approach with stents often placed across the ampulla.[8] Consequently, many patients develop cholangitis requiring hospital readmissions and reinterventions. Moreover, pancreatitis is another serious complication of EBD.[9]

Percutaneous biliary drainage (PBD) is the alternative approach to EBD and is often used when EBD is unsuccessful

☆ This study was reviewed and approved by the ethics committee of the Erasmus MC Cancer Institute on May 25, 2020 (MEC–2019-0789). All human participants gave written informed consent. Trial registration number: NL9624 (ICTRP).

* Corresponding author. Address: Department of Surgery, Erasmus MC Cancer Institute, Rotterdam, The Netherlands. Tel.: +31(0)107031810.
E-mail address: b.grootkoerkamp@erasmusmc.nl (B. Groot Koerkamp).
† These authors share second authorship.
‡ Name is deceased

https://doi.org/10.1016/j.jhepr.2025.101541

or unfeasible (Fig. 1B).[10,11] PBD typically involves an internalized drain with the tip of the drain beyond both the tumor and the ampulla into the duodenum. The downside of an internalized percutaneous biliary drain is the same as for an endoscopic stent; the ampulla is crossed, and bacterial colonization of the bile ducts occurs.[12] Nasobiliary drainage (NBD) is an alternative endoscopic approach that is commonly used in Asia (Fig. 1C), but in this technique, the ampulla is disrupted.[13]

All three approaches of biliary drainage for MHBO entail a high risk of cholangitis resulting in clinical deterioration, multiple reinterventions, and a mortality up to 36% within 90 days after drainage.[14,15] Consequently, only 13% of all patients with advanced pCCA received palliative systemic treatment in a nationwide study in the Netherlands, a country with centralized care for primary liver tumors and universal health care coverage.[4]

Primary percutaneous stenting (PPS) with uncovered SEMS that do not cross the ampulla is an alternative approach that aims to prevent bacterial colonization of the bile ducts. After stent placement, the puncture tract is sealed immediately without the need for an external drain (Fig. 1D). The aim of this study was to investigate drainage-related severe complications and the proportion of patients receiving palliative systemic treatment after PPS in palliative patients with MHBO.

Materials and methods

Study design

The TESLA trial is a single-arm, single-center investigator-initiated phase II trial. The study (NL9624, ICTRP) was reviewed and approved by the institutional review board of the Erasmus MC (Rotterdam, The Netherlands). The trial started as a proof-of-concept pilot with 10 patients, after which the cohort was expanded to 67 patients.

Study population and inclusion criteria

Patients with a high suspicion of MHBO who could not undergo a curative-intent resection or liver transplantation were eligible for inclusion. All patients had metastatic disease, locally advanced disease, and/or a poor performance status (*i.e.* Eastern Cooperative Oncology Group [ECOG] performance scale 2 or higher). Eligibility was determined during the multidisciplinary team meeting. All patients presented with symptomatic hyperbilirubinemia (serum total bilirubin level >3.0 mg/dl). The definitive diagnosis of MHBO was established based on percutaneous biopsy of the primary tumor or metastasis, endoscopic ultrasound-guided fine-needle aspiration or biopsy (EUS-FNA/B), brush cytology obtained during PPS, or follow-up. An Endoscopic retrograde cholangiopancreatography (ERCP) for a biopsy or brush cytology was never performed. Next-generation sequencing of the brush was performed if cytology results were inconclusive. In the absence of pathological confirmation before biliary drainage, patients were eligible if MHBO was considered very likely (*i.e.* >90% certainty), based on clinical symptoms, laboratory tests (including serum IgG4, CA 19-9, and trends in bilirubin level), and radiological imaging.[16] This was determined by the multidisciplinary hepatobiliary team. Informed consent was obtained at the outpatient clinic.

Exclusion criteria

Patients who had undergone previous EBD or PBD procedures were ineligible. Patients with a malignant distal biliary obstruction (*i.e.* with involvement of the ampulla on imaging) were excluded. In addition, patients with a clinical presentation of cholangitis before PPS were excluded.[17] Patients were ineligible if they had a spontaneous drop in total bilirubin level before biliary drainage, which could indicate a benign cause (*e.g.* autoimmune cholangitis [AIC]).

The extent of proximal biliary isolation and a life-expectancy of less than 3 months were no exclusion criteria.

Study intervention

PPS was performed under conscious sedation with propofol and anesthetic monitoring. An interventional radiologist performed the biliary drainage procedure. Ultrasound and fluoroscopy were used to access the biliary tree with a right intercostal and/or left epigastric percutaneous approach. The

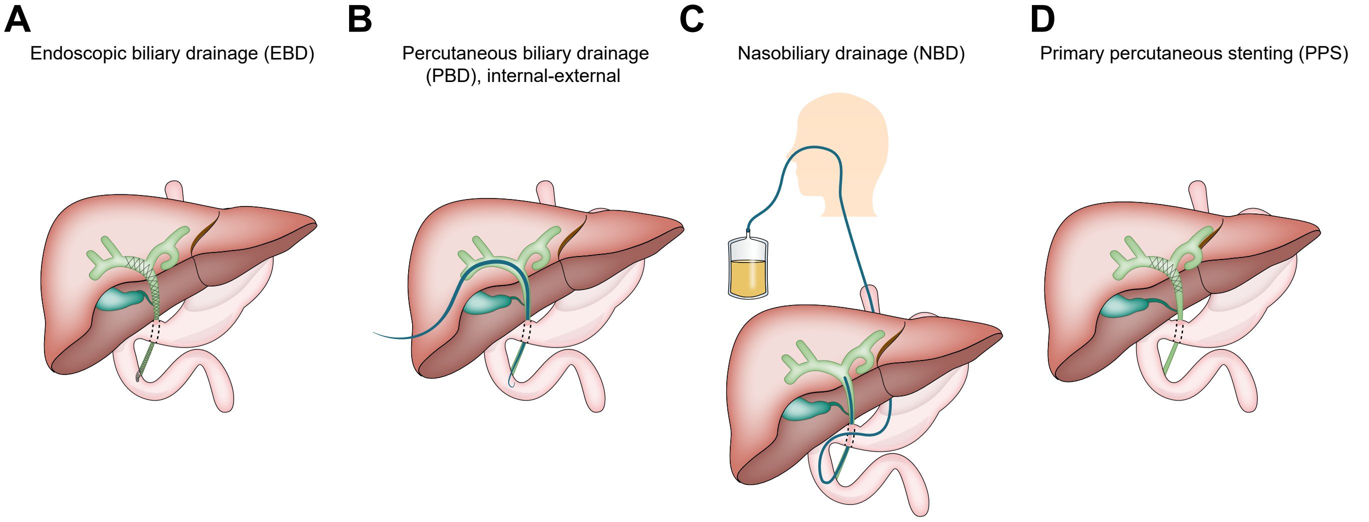

Fig. 1. Different biliary drainage approaches. (A) Endoscopic biliary drainage; (B) *percutaneous biliary drainage, internal–external*; (C) nasobiliary drainage; and (D) primary percutaneous stenting.

aim was to drain at least 50% of the total liver volume. One to three uncovered SEMS were placed without cannulation of the ampulla. Liver segments with atrophy or portal vein occlusion were not drained. For a detailed description of the study intervention, see Supplementary methods.

Outcomes and definitions

The primary outcomes of the initial pilot with 10 patients were safety and feasibility. Safety was defined as low (<20%) drainage-related 90-day mortality. Feasibility was defined as primary technical success in >70% of patients. The primary outcome of the full cohort was the proportion of 90-day drainage-related severe complications. This included cholangitis, acute cholecystitis, acute pancreatitis, biliary leakage, hemorrhage, and persistent jaundice (see Table S1 for definitions). Secondary outcomes included primary and secondary technical success of stent placement at the initial drainage procedure, the number of reinterventions, hospital admission days after drainage, bile cultures at initial drainage, and primary successful drainage. Primary technical success was defined as successful passage of the tumor and stent placement without the necessity for an external drain during the initial drainage procedure. Secondary technical success was defined as successful passage of the tumor and stent placement during a second attempt 4–7 days after an unsuccessful initial procedure. Primary successful drainage was defined as a bilirubin level below 3.0 mg/dl or a reduction in bilirubin level of at least 50% within 14 days after the first drainage procedure. Other secondary outcomes were the proportion of patients who started with palliative systemic treatment, 90-day mortality, and OS.

Statistical analysis

For dichotomous and categorical data, proportions and 95% CIs were calculated. The median and IQR were reported for continuous variables. Survival outcomes were estimated using the Kaplan–Meier method. A prespecified subgroup survival analysis was performed for the type of malignancy.

Results

From October 2020 until June 2023, 99 patients with a high suspicion of MHBO who were ineligible for surgical resection presented at Erasmus MC Cancer Center. Thirty patients (30.3%) were excluded; eight patients with pCCA were eligible for the liver transplant protocol, seven patients were referred after EBD at the referring center, 11 patients underwent EBD at the discretion of the treating gastroenterologist during the pilot phase of the trial, two patients had a spontaneous decrease in serum total bilirubin level, and two patients developed spontaneous cholangitis before biliary drainage.

All eligible 69 patients gave written informed consent. Two patients were excluded after informed consent because of a low suspicion of MHBO during percutaneous cholangiography without stent placement or biliary drainage. Both patients had non-obstructive jaundice and died within a few weeks because of liver failure. All remaining 67 patients underwent PPS and were analyzed (Fig. 2). Baseline patient and tumor characteristics are shown in Table 1. The median age was 70 years, and 14 patients (20.9%) were ≥80 years old. The cause of MHBO was pCCA in 27 patients (40.3%), iCCA in 23 patients (34.3%), GBC in nine

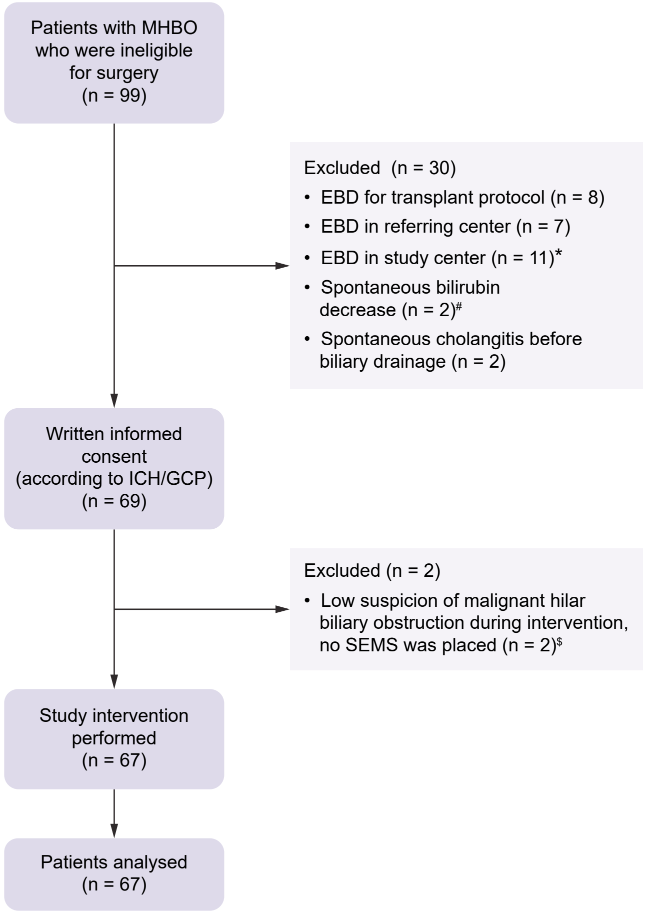

Fig. 2. Flowchart. EBD, endoscopic biliary drainage; MHBO, malignant hilar biliary obstruction; SEMS, self-expandable metal stents; ICH/GCP, International Council for Harmonisation/Good Clinical Practice.

patients (13.4%), liver metastases in seven patients (10.5%), and hepatocellular carcinoma in one patient (1.5%). Patients were ineligible for resection because of metastatic disease in 30 patients (44.8%), locally advanced disease in 28 patients (41.8%), and poor performance status in nine patients (13.4%).

Safety and feasibility

The drainage-related 90-day mortality rate (*i.e.* safety) was 0% in both the initial pilot of 10 patients and the full cohort. The chance of primary technical success (*i.e.* feasibility) was 90.0% (9/10) in the initial pilot and 98.5% (66/67) in the full cohort. In only one patient (1.5%), the tumor could not be passed with a wire during the first drainage procedure. Therefore, an external biliary drain was placed without crossing the ampulla. Five days later, a wire could be successfully passed beyond the tumor, followed by stenting and immediate removal of the external biliary drain with sealing of the puncture tract. In 19 patients (28.3%), only one SEMS was placed at the initial drainage procedure, two SEMS were placed in 41 patients (61.2%), and three SEMS were placed in seven patients (10.4%).

Drainage-related complications

Twelve patients (17.9%) had drainage-related complications within 90 days (Table 2). Two patients developed cholecystitis

Table 1. Baseline characteristics.

Characteristics	Primary percutaneous stenting (n = 67), n (%)/median (IQR)
Age (years)	70 (64–78)
Female	38 (56.7)
BMI	24 (22–28)
ASA score	
2	16 (23.9)
3	45 (67.1)
4	6 (9.0)
Total bilirubin before drainage (mg/dl)	15.1 (9.1–20.5)
Type of malignancy	
Perihilar cholangiocarcinoma	
pCCA	27 (40.3)
Intrahepatic cholangiocarcinoma	23 (34.3)
Gallbladder carcinoma	9 (13.4)
Hepatocellular carcinoma	1 (1.5)
Liver metastasis	7 (10.5)
Colorectal cancer	4 (6.0)
Pancreatic cancer	2 (3.0)
Ovarian cancer	1 (1.5)
Bismuth–Corlette (on imaging, only for pCCA, including M1 patients)	
1	0 (0.0)
2	6 (22.2)
3a	8 (29.7)
3b	3 (11.1)
4	10 (37.0)
Stage of disease	
Technically resectable, but poor performance status (ECOG ≥2)	9 (13.4)
Locally advanced	28 (41.8)
Metastatic	30 (44.8)

ECOG, Eastern Cooperative Oncology Group; pCCA, perihilar cholangiocarcinoma; ASA, American Society of Anesthesiologists; BMI, body mass index.

Table 2. Primary and secondary outcomes within 90 days.

Outcomes	Primary percutaneous stenting (n = 67), n (%)/median (IQR)
**Primary outcome**	
Drainage-related complication*	12 (17.9)
Cholangitis	
After initial drainage	0 (0.0)
After reintervention	1 (1.5)†
Acute cholecystitis	2 (3.0)
Acute pancreatitis	0 (0.0)
Biliary leak	1 (1.5)
Hemorrhage	3 (4.5)
Persistent jaundice	6 (9.0)
**Secondary outcomes**	
Primary successful drainage	61 (91.0)
Secondary successful drainage	67 (100)
Reinterventions	
Non-biliary reinterventions	2 (3.0)
Biliary reinterventions within 90 days	9 (13.4)
Biliary reinterventions after 90 days	16 (23.9)
Admission days after drainage	1 (1–2)
Readmission	6 (9.0)
Total admission days in 90 days	1 (1–3)
Microorganisms in bile culture‡	2 (3.0)
Total bilirubin after 14 days (mg/dl)	3.5 (2.2–5.6)
Absolute bilirubin decrease after 14 days (mg/dl)	10.5 (6.5–15.8)
Relative bilirubin decrease after 14 days (%)	73 (65–80)
Palliative systemic treatment	42 (62.7)
90-day mortality	12 (17.9)
Overall survival (months), median (95% CI)	10.1 (7.9–14.4)

*The total number of patients with a drainage-related complications is 12, because one patient had 2 complications.
†This patient had pCCA with extension of the biliary disease up to the ampulla and required percutaneous transpapillary stent extension. After this reintervention, she developed cholangitis that successfully resolved with intravenous antibiotics.
‡*Raoultella ornithinolytica* and *Streptococcus parasanguinis*. pCCA, perihilar cholangiocarcinoma.

within 1 week after initial biliary drainage; one underwent a laparoscopic cholecystectomy, and one underwent percutaneous drainage of the gallbladder, both without complications. One patient (1.5%) had a hilar biliary leak during the initial PPS, which resolved with an external biliary drain that was removed after 1 week. Three patients (4.5%) had hemorrhage from (branches of) the portal vein or hepatic artery; all required blood transfusion, and two patients underwent a reintervention with coiling of an arterial branch. Sixty-one patients (91.0%) were successfully drained after the first drainage procedure. Six patients (9.0%) had persistent jaundice (*i.e.* bilirubin level <3.0 mg/dl or drop >50% after 14 days) after initial PPS, of whom five had very large intrahepatic tumors that required a reintervention with additional percutaneous SEMS after a median of 31 days (range 7–68 days). In all five patients, bilirubin levels normalized after additional drainage without further complications. The other patient had pCCA with extension of the biliary disease up to the ampulla and required percutaneous transpapillary stent extension. After this reintervention, she developed cholangitis that resolved with intravenous antibiotics. None of the patients underwent additional EBD. No pancreatitis or cholangitis was seen after the initial PPS.

Bilirubin levels and bile cultures

Sixty-one patients (91.0%) reached primary successful drainage (*i.e.* bilirubin levels <3.0 mg/dl or a reduction in bilirubin level of at least 50% within 14 days). The median total bilirubin level at baseline before PPS was 15.1 mg/dl (IQR 9.1–20.5 mg/dl). After 2 weeks, the median bilirubin level was 3.5 mg/dl (IQR 2.2–5.6 mg/dl). Within 2 weeks, the median absolute decrease in total bilirubin level was 10.8 mg/dl (IQR 6.5–15.8 mg/dl), and the median relative decrease was 73% (IQR 65–80%). Fifty-five of 67 patients (82%) achieved bilirubin levels below 3 mg/dl within 28 days after biliary drainage. Of the 12 patients with bilirubin levels above 3 mg/dl, 7 had a >50% reduction in bilirubin levels within 14 days after biliary drainage. The bilirubin level over time of each patient is presented in Fig. S1.

In 65 patients (97.0%), no microorganisms were cultured in the bile specimens collected during the initial drainage procedure. Two patients (3.0%) had positive bile cultures (*Raoultella ornithinolytica* and *Streptococcus parasanguinis*) but never had clinical signs of cholangitis.

Pathological confirmation

Pathological confirmation of malignancy was obtained in 59 of 67 patients (88.1%). In nine patients (13.4%), pathological confirmation of malignancy had been obtained before jaundice and study inclusion. In patients with a diagnosis other than pCCA, a percutaneous biopsy of the primary tumor was performed during the drainage procedure in 17 patients (25.4%). Sixteen (94.1%) of these biopsies confirmed malignancy. Percutaneous biopsy of a peritoneal metastasis was performed in four patients (6.0%), and all confirmed malignancy. Cytology of a percutaneous biliary brush was performed in 43

patients, of whom 67% (29/43) showed at least a high suspicion of malignancy (*i.e.* WHO 6 or 7[18]). EBD with brush or biopsy was never performed.

Eight patients (11.9%) had no pathological confirmation of cancer, of whom three died from progressive disease confirmed on imaging. Of the remaining five patients (7.5%), four were octogenarians with a high suspicion for pCCA without signs or symptoms of AIC or liver flukes. They did not want any treatment other than biliary drainage. The other patient showed no progression of disease on imaging during follow-up after 21 months. These five patients are still alive after a median follow-up of 15.9 months (range 9.6–21.3 months), without long-term complications from the uncovered SEMS.

Palliative treatment

Forty-two patients (62.7%) received palliative systemic treatment, all starting within 4 weeks of PPS. Nine patients (13.4%) experienced rapidly progressive disease and died within 90 days before starting treatment; five patients presented with metastatic disease with ascites, and four had rapid progression of large multifocal iCCA. Four patients (6.0%) were ineligible for systemic treatment because of comorbidities. Seven patients (10.4%), who were all octogenarians, decided not to have systemic treatment. The remaining five patients (7.5%), including four octogenarians, had no pathological confirmation of cancer and were therefore ineligible for palliative systemic treatment. Palliative systemic treatment was never withheld because of inadequate biliary drainage or drainage-related complications. After excluding patients with rapidly progressive disease, octogenarians, and patients without pathological confirmation, all but one patient (98%) received palliative treatment.

Recurrent jaundice

Eighteen patients (26.9%) presented with recurrent jaundice after more than 90 days. Jaundice recurred after a median of 10.4 months (IQR 5.7–13.8 months). Recurrent jaundice developed in six of 27 patients (22.2%) with pCCA and in 12 of 40 patients (30.0%) with a diagnosis other than pCCA (p = 0.68). Nine of 19 patients (47.3%) with only one initial stent developed recurrent jaundice *vs*. 14 of 48 patients (29.2%) with multiple stents (p = 0.26).

Sixteen of 18 patients (88.9%) who presented with recurrent jaundice underwent a biliary reintervention with technically successful percutaneous additional stent placement without crossing the ampulla and without leaving an external drain. The cause of recurrent jaundice was progression of the disease with obstruction of undrained segments in 12 of 16 patients (75%), stent occlusion caused by tumor ingrowth on imaging in three (of whom none had pCCA), and stent occlusion caused by sludge in one patient. The remaining two patients (3.0%) declined a reintervention and died because of disease progression. None of the patients with recurrent jaundice developed cholangitis.

Survival

The median OS was 10.1 months (95% CI 7.9–14.4 months), with a 6-month OS of 68.7% (95% CI 58.4–80.7%) (Fig. 3).

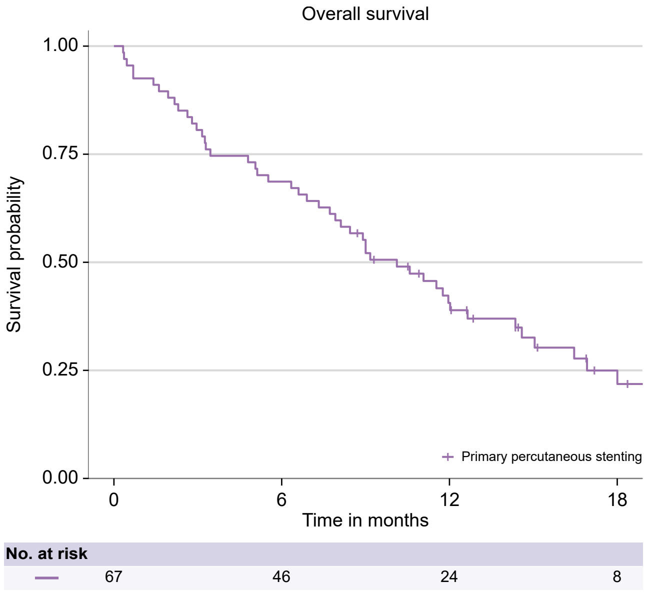

Fig. 3. Overall survival.

After excluding the five patients with a high suspicion but no pathological confirmation of cancer, the median OS was similar (9.0 months, p = 0.94). The median OS was 11.5 months (95% CI 8.1 to not reached) for patients with pCCA *vs*. 9.2 months (95% CI 6.3–15.0 months) for other malignancies (p = 0.46).

Follow-up data were complete for all 67 patients. Forty-eight patients (71.6%) died during follow-up, and the median follow-up of patients alive at last follow-up was 17.2 months (95% CI 12.9–19.7 months). Twelve patients (17.9%) died within 90 days after the initial primary percutaneous drainage; 11 patients (14.9%) died because of rapid progression of peritoneal disease (n = 4) or extensive liver disease (n = 7). The other patient was a 78-year-old male who died because of acute renal failure caused by severe hyperbilirubinemia at presentation with bile cast nephropathy that did not recover after adequate biliary drainage. The 90-day mortality rate was 7.5% in patients with pCCA (2/27) *vs*. 25.0% in patients with other malignancies (10/40) (p = 0.12). Mortality within 30 days was 7.5%. No patients died from drainage-related complications or biliary sepsis.

Discussion

This investigator-initiated phase II trial of 67 patients demonstrated that PPS in patients with unresectable MHBO resulted in a low risk of drainage-related severe complications of 18% and a drainage-related 90-day mortality of 0%. Reinterventions resolved all complications. The 90-day mortality rate was 18% and, except for one patient with renal failure, was caused by rapid disease progression. Cholangitis and acute pancreatitis were never observed after initial PPS. Most patients (63%) started with palliative systemic treatment within 4 weeks after drainage. Complications of drainage or inadequate drainage never precluded palliative treatment. These outcomes of PPS are a considerable improvement compared with historical cohorts using EBD or conventional internal–external PBD (Table 3). PPS resolved both drawbacks of conventional

Table 3. Comparison of historical cohorts of patients with pCCA or MHBO using EBD or PBD.

Author	Study design	Setting	Type of biliary drainage	Number of patients	90-day mortality (%)	Drainage-related complications (%)	Cholangitis (%)	Pancreatitis (%)
Coelen *et al.*[17]	Prospective RCT	pCCA (resectable)	EBD	27	11	67	37	19
			PBD	27	41	63	59	4
Kloek *et al.*[29]	Retrospective, single center	pCCA (resectable)	EBD	90	NR	82	48	8
			PBD	11	NR	45	9	0
Elmunzer *et al.*[22]	Prospective RCT	MHBO (any)	EBD	8	50	75	NR	NR
			PBD	5	80	80	NR	NR
Rees *et al.*[30]	Retrospective, multicenter	Any biliary obstruction (palliative)	PBD	16.822	23*	20	11	NR
Paik *et al.*[28]	Retrospective, multicenter	pCCA (palliative)	EBD	44	NR	30	30	0
			PBD	41	NR	32	22	5
Lubbe *et al.*[27]	Retrospective, multicenter	MHBO (palliative)	EBD	117	NR	45	21	9
			PBD	146	NR	56	25	0
Van Keulen *et al.*[14]	Retrospective, single center	pCCA (palliative)	EBD	186	36†	12†	6	3
			PBD	25			8	0
Liang *et al.*[26]	Retrospective, single center	MHBO (palliative)	EBD	126	NR	39	28	12
			PBD	80	NR	40	29	0
**Franssen *et al.* (this study)**	**Prospective single arm**	**MHBO (palliative)**	**PPS**	**67**	**18**	**18**	**2**	**0**

Bold text represents key findings from this study.
*30-day mortality.
†Same in both types of biliary drainage. EBD, endoscopic biliary drainage; MHBO, malignant hilar biliary obstruction; NR, not reported; PBD, percutaneous biliary drainage; pCCA, perihilar cholangiocarcinoma; PPS, primary percutaneous stenting; RCT, randomized controlled trial.

external and/or internal PBD; the stents do not cross the ampulla, and no external drain is used.

This is the first prospective study investigating PPS for MHBO. In a retrospective study, Thornton *et al.* analyzed 71 patients receiving PPS at Memorial Sloan Kettering Cancer Center (MSKCC).[19] Most patients (86%), however, did not have a MHBO but a distal biliary obstruction. Moreover, most patients (96%) had a temporary external biliary catheter. Similar to the present study, major drainage-related complications occurred in 14%. PPS is still the preferred approach at MSKCC for MHBO in the palliative setting.[20]

Guidelines are inconsistent about the optimal approach for palliative drainage of MHBO. The clinical guideline of the American Society for Gastrointestinal Endoscopy (ASGE 2021) recommends EBD or PBD: ‘the standard of care for the treatment of unresectable MHBO is based on patient preferences, disease characteristics, and local expertise’.[6] The NCCN guideline for biliary cancer does not specify a preference for EBD or PBD.[21] In these guidelines, PBD involves internal–external PBD. PPS in the present study is also a percutaneous approach but differs because of primary stent placement without crossing the ampulla and without leaving an external biliary drain.

The only two randomized controlled trials (RCTs) comparing EBD *vs.* PBD for MHBO were both prematurely closed. They showed similar poor outcomes for both approaches. The DRAINAGE trial in the Netherlands included patients with potentially resectable pCCA. It was discontinued by the data monitoring committee because of increased mortality in the PBD arm that was not statistically significant.[17] The INTERCPT trial in the USA included patients with suspected MHBO, regardless of tumor stage.[22] It was discontinued because of slow accrual. Regardless of the approach (EBD or PBD), the 90-day mortality after biliary drainage was very high: 26% (11 of 42 patients) in the DRAINAGE trial and 62% (8 of 13 patients) in the INTERCPT trial. Table 3 also shows similar poor outcomes of several non-randomized cohorts of patients with MHBO undergoing EBD or internal–external PBD.

In the present study of consecutive patients in a tertiary referral center, pCCA caused MHBO in only 40% of patients. MBHO was caused by iCCA in 34%, GBC in 13%, and liver metastases in 10%. The differential diagnosis of MHBO includes benign causes, particularly AIC. Ruling out AIC can be difficult because not all patients with AIC have an elevated serum IgG4. Moreover, a biopsy to demonstrate elevated $IgG4^{+}$ plasma cells is typically challenging to obtain because a mass is rarely seen on imaging. Both endoscopists and interventional radiologists are concerned about the long-term sequelae of placing an uncovered SEMS in the bile duct that cannot be removed in the event of benign disease.[23] In the present study, all patients had a very high clinical suspicion of cancer based on high bilirubin levels at baseline without evidence of IgG4-associated cholangiopathy.[24] Only five patients (7.5%) are alive without confirmation of cancer, of whom four are octogenarians preferring best supportive care.

Guidelines increasingly recommend placement of multiple stents to drain more than 50% of the total liver volume.[6,7] A recent retrospective study showed that in patients with inoperable pCCA, who survive at least 6 months, endoscopic placement of SEMS leads to fewer reinterventions in comparison with endoscopic plastic stents.[25] Guidelines also

agree that atrophic segments or segments with portal vein occlusion should not be drained. We recommend draining at least 50% of the normal liver with a low threshold to place multiple SEMS.

In the present study, no patients developed cholangitis after initial primary percutaneous stenting. Previous studies have reported an incidence of cholangitis after EBD varying between 6% and 30% in patients with unresectable pCCA or MHBO (Table 3).[14,26–28] Retrospective studies likely underestimated the true risk of cholangitis. The absence of cholangitis after initial PPS in all 67 patients is the main advantage of PPS. The lower risk of drainage-related complications after PPS, with the absence of cholangitis and pancreatitis, resulted in improved OS, both short and long terms. Therefore, improving biliary drainage should be the primary focus of multidisciplinary teams treating patients with biliary cancer.

This study has several limitations. First, most PPS procedures have been performed by a single interventional radiologist with extensive expertise in biliary interventions. Second, the present study is a non-randomized study, and therefore, the results can only be compared with historical cohorts. A multicenter randomized controlled trial (NCT06671418) is ongoing to investigate the promising results of the present study with PPS in comparison with the current standard of care (*i.e.* EBD). Third, the biliary drainage technique in this study requires referral to an expert center for primary liver tumors before any drainage attempt. Guidelines, however, already recommend that patients with primary liver tumors are discussed in a multidisciplinary team at an expert center before biliary drainage.[21]

In conclusion, PPS for palliative patients with MHBO had a low incidence of drainage-related complications without cholangitis or pancreatitis after the initial stenting. Most patients (61.2%) started with palliative systemic treatment. These results compare very favorably to both EBD and internal and/or external PBD. Novel targeted treatments and immunotherapy for biliary cancer can benefit patients only after adequate biliary drainage without cholangitis.

Affiliations

[1]*Department of Surgery, Erasmus MC Cancer Institute, Rotterdam, The Netherlands;* [2]*Department of Gastroenterology and Hepatology, Erasmus MC Cancer Institute, Rotterdam, The Netherlands;* [3]*Department of Pathology, Erasmus MC Cancer Institute, Rotterdam, The Netherlands;* [4]*Department of Medical Oncology, Erasmus MC Cancer Institute, Rotterdam, The Netherlands;* [5]*Department of Radiology, Erasmus MC Cancer Institute, Rotterdam, The Netherlands*

Abbreviations

AIC, autoimmune cholangitis; ASGE, American Society for Gastrointestinal Endoscopy; EBD, endoscopic biliary drainage; ECOG, Eastern Cooperative Oncology Group; EUS-FNA/B, endoscopic ultrasound-guided fine-needle aspiration or biopsy; GBC, gallbladder cancer; iCCA, intrahepatic cholangiocarcinoma; MHBO, malignant hilar biliary obstruction; MSKCC, Memorial Sloan Kettering Cancer Center; NBD, nasobiliary drainage; OS, overall survival; PBD, percutaneous biliary drainage; pCCA, perihilar cholangiocarcinoma; PPS, primary percutaneous stenting; SEMS, self-expandable metal stent.

Financial support

The authors did not receive any financial support to produce this manuscript.

Conflicts of interest

The authors of this study declare that they do not have any conflict of interest.

Please refer to the accompanying ICMJE disclosure forms for further details.

Authors' contributions

Concept and design: SF, VvV, LvD, BGK. Methodology: VvV, AM, BGK. Formal analysis: SF, MR, BGK. Investigation: SF, MR, MD, LvD, BH, DB, AM. Writing—original draft: SF, MR. Writing—review and editing: VvV, MB, MD, LvD, MH, RdW, JdJ, WP, RP, BGK. Visualization: SF, MR, VvV, BGK. Project administration: SF, MR, BGK. Supervision: VvV, RP, AM, BGK.

Data availability

The data supporting the findings of this study are available upon reasonable request from the corresponding author. Due to privacy and ethical restrictions, individual participant data cannot be shared publicly.

Supplementary data

Supplementary data to this article can be found online at https://doi.org/10.1016/j.jhepr.2025.101541.

References

[1] Alvaro D, Bragazzi MC, Benedetti A, et al. Cholangiocarcinoma in Italy: a national survey on clinical characteristics, diagnostic modalities and treatment. Results from the "Cholangiocarcinoma" committee of the Italian Association for the Study of Liver disease. Dig Liver Dis 2011;43:60–65.

[2] Hawkins WG, DeMatteo RP, Jarnagin WR, et al. Jaundice predicts advanced disease and early mortality in patients with gallbladder cancer. Ann Surg Oncol 2004;11:310–315.

[3] Chaiteerakij R, Harmsen WS, Marrero CR, et al. A new clinically based staging system for perihilar cholangiocarcinoma. Am J Gastroenterol 2014;109:1881–1890.

[4] van Keulen AM, Franssen S, van der Geest LG, et al. Nationwide treatment and outcomes of perihilar cholangiocarcinoma. Liver Int 2021;41:1945–1953.

[5] Blechacz B. Cholangiocarcinoma: current knowledge and new developments. Gut Liver 2017;11:13–26.

[6] Qumseya BJ, Jamil LH, Elmunzer BJ, et al. ASGE guideline on the role of endoscopy in the management of malignant hilar obstruction. Gastrointest Endosc 2021;94:222. 34.e222.

[7] Vogel A, Bridgewater J, Edeline J, et al. Biliary tract cancer: ESMO Clinical Practice Guideline for diagnosis, treatment and follow-up. Ann Oncol 2023;34:127–140.

[8] Avisse C, Flament JB, Delattre JF. Ampulla of Vater. Anatomic, embryologic, and surgical aspects. Surg Clin North Am 2000;80:201–212.

[9] Ding X, Zhang F, Wang Y. Risk factors for post-ERCP pancreatitis: a systematic review and meta-analysis. Surgeon 2015;13:218–229.

[10] Perez-Johnston R, Deipolyi AR, Covey AM. Percutaneous biliary interventions. Gastroenterol Clin North Am 2018;47:621–641.

[11] Wiggers JK, Groot Koerkamp B, Coelen RJ, et al. Preoperative biliary drainage in perihilar cholangiocarcinoma: identifying patients who require percutaneous drainage after failed endoscopic drainage. Endoscopy 2015;47:1124–1131.

[12] Rösch T, Triptrap A, Born P, et al. Bacteriobilia in percutaneous transhepatic biliary drainage: occurrence over time and clinical sequelae. A prospective observational study. Scand J Gastroenterol 2003;38:1162–1168.

[13] Kawakami H, Kuwatani M, Onodera M, et al. Endoscopic nasobiliary drainage is the most suitable preoperative biliary drainage method in the management of patients with hilar cholangiocarcinoma. J Gastroenterol 2011;46:242–248.

[14] Keulen AV, Gaspersz MP, van Vugt JLA, et al. Success, complication, and mortality rates of initial biliary drainage in patients with unresectable perihilar cholangiocarcinoma. Surgery 2022;172:1606–1613.

[15] Moole H, Dharmapuri S, Duvvuri A, et al. Endoscopic versus percutaneous biliary drainage in palliation of advanced malignant hilar obstruction: a meta-analysis and systematic review. Can J Gastroenterol Hepatol 2016;2016:4726078.

[16] Coelen RJS, Gaspersz MP, Labeur TA, et al. Validation of the Mayo Clinic staging system in determining prognoses of patients with perihilar cholangiocarcinoma. Clin Gastroenterol Hepatol 2017;15:1930. 9.e1933.

[17] Coelen RJS, Roos E, Wiggers JK, et al. Endoscopic versus percutaneous biliary drainage in patients with resectable perihilar cholangiocarcinoma: a multicentre, randomised controlled trial. Lancet Gastroenterol Hepatol 2018;3:681–690.
[18] Pitman MB, Centeno BA, Reid MD, et al. The World Health Organization reporting system for pancreaticobiliary cytopathology. Acta Cytol 2023;67:304–320.
[19] Thornton RH, Frank BS, Covey AM, et al. Catheter-free survival after primary percutaneous stenting of malignant bile duct obstruction. AJR Am J Roentgenol 2011;197:W514–W518.
[20] Deipolyi AR, Covey AM. Palliative percutaneous biliary interventions in malignant high bile duct obstruction. Semin Intervent Radiol 2017;34:361–368.
[21] Benson AB, D'Angelica MI, Abbott DE, et al. Hepatobiliary cancers, version 2.2021, NCCN clinical practice guidelines in Oncology. J Natl Compr Canc Netw 2021;19:541–565.
[22] Elmunzer BJ, Smith ZL, Tarnasky P, et al. An unsuccessful randomized trial of percutaneous vs endoscopic drainage of suspected malignant hilar obstruction. Clin Gastroenterol Hepatol 2021;19:1282–1284.
[23] Haseeb A, Amateau SK. Metal biliary stents in benign disease. Clin Gastroenterol Hepatol 2019;17:1029–1032.
[24] Hubers LM, Beuers U. How to diagnose immunoglobulin G4-associated cholangitis: the jack-of-all-trades in the biliary tract. Viszeralmedizin 2015;31:185–188.
[25] Fritzsche JA, de Jong DM, Borremans JJMM, et al. Long-term efficacy of metal versus plastic stents in inoperable perihilar cholangiocarcinoma; a multicenter retrospective propensity score matched comparison. HPB 2023;25:798–806.
[26] Liang XY, Li W, Liu F, et al. A retrospective study of biliary drainage strategies for patients with malignant hilar biliary strictures. Cancer Manag Res 2021;13:4767–4776.
[27] Lubbe J, Lindemann J, Gondo W, et al. Endoscopic versus percutaneous intervention for palliation in malignant hilar bile duct obstruction—a comparative cohort study. HPB (Oxford) 2022;24:2145–2156.
[28] Paik WH, Park YS, Hwang JH, et al. Palliative treatment with self-expandable metallic stents in patients with advanced type III or IV hilar cholangiocarcinoma: a percutaneous versus endoscopic approach. Gastrointest Endosc 2009;69:55–62.
[29] Kloek JJ, van der Gaag NA, Aziz Y, et al. Endoscopic and percutaneous preoperative biliary drainage in patients with suspected hilar cholangiocarcinoma. J Gastrointest Surg 2010;14:119–125.
[30] Rees J, Mytton J, Evison F, et al. The outcomes of biliary drainage by percutaneous transhepatic cholangiography for the palliation of malignant biliary obstruction in England between 2001 and 2014: a retrospective cohort study. BMJ Open 2020;10:e033576.

Keywords: Cholangiocarcinoma; Jaundice; Self-expanding metal stent.
Received 14 April 2025; received in revised form 19 June 2025; accepted 24 July 2025; Available online 11 September 2025

Journal of Hepatology, Volume *7*

Supplemental information

Primary percutaneous stenting for palliative biliary drainage of patients with malignant hilar biliary obstruction: TESLA trial

Stijn Franssen, Merve Rousian, Victorien van Verschuer, Marco Bruno, Michail Doukas, Lydi van Driel, Marjolein Homs, Behnam Mohseny, Roeland de Wilde, Jeroen de Jonge, Wojciech Polak, Robert Porte, Diederik Bijdevaate, Adriaan Moelker, and Bas Groot Koerkamp

Primary percutaneous stenting for palliative biliary drainage of patients with malignant hilar biliary obstruction: TESLA trial☆

Stijn Franssen, Merve Rousian, Victorien van Verschuer, Marco Bruno, Michail Doukas, Lydi van Driel, Marjolein Homs, Behnam Mohseny, Roeland de Wilde, Jeroen de Jonge, Wojciech Polak, Robert Porte, Diederik Bijdevaate, Adriaan Moelker, Bas Groot Koerkamp

Table of contents

Supplementary methods……………………………………………………………………2

Fig. S1……………………………………………………………………………………3

Table S1…………………………………………………………………………………4

Supplementary methods. Detailed description of study intervention.

The percutaneous transhepatic bile duct puncture was performed with an 18G or 21G Chiba needle. Then, a 4 or 5 Fr sheath (e.g., Neffset, Cook Medical, Bloomington, IN)) was placed and a 0.032-0.038 inch guidewire with 4 Fr catheter was advanced beyond the bile duct stricture or obstruction without passing the ampulla. After crossing the stricture, the guidewire was preferably replaced by a stiff Amplatz guidewire. The sheath was replaced by a 7 or 8 Fr sheath allowing for a brush and stent placement. Brush cytology was performed by moving the brush five times back and forth through the stricture. This was repeated with a second brush to increase the diagnostic yield. Before stent insertion, the stricture was dilated with a balloon catheter that was pushed over the guidewire (diameter 6 to 8 mm). Next, a biliary stent (Hilzo biliary stent, uncovered straight type, 8 or10 mm diameter and 6 or 8 cm length) was inserted, again without advancing the wire beyond the ampulla. The puncture tract was sealed with 0.5 gr Avitene (Becton Dickinson NJ, USA) mixed with 8 ml of iodine contrast agent to visualize the sealing track. No external biliary drain was left after stent placement. Crossing of the ampulla with a drain or stent (i.e., internal drainage) was avoided.

When multiple stents were placed, a guidewire was inserted in 2 or 3 segments before balloon dilatation and stent placement. Multiple stents were mostly placed in a “kissing” fashion (i.e., adjacent in the common hepatic bile duct), rather than in a fenestrated fashion. Advancing the guidewire beyond the tumor can be challenging and may require several attempts. Whenever the tumor could not be passed during the procedure, an external biliary drain was placed. A second attempt of stenting was performed after 4 to 7 days.

All patients received antibiotics before and after biliary drainage. All patients received a one-time preprocedural intravenous dose of cefuroxime 1500mg and metronidazole 500mg and 5 days of oral amoxicillin/clavulanic acid 3dd 625mg after the procedure, or ciprofloxacin 2dd 500mg in patients with a penicillin allergy. Patients who presented with a bilirubin above 15.0 mg/dL were already started on the same oral antibiotics prior to the procedure to minimize the risk of spontaneous cholangitis before biliary drainage.

Fig. S1. Serum bilirubin level curve of all patients in the first 28 days after PPS.

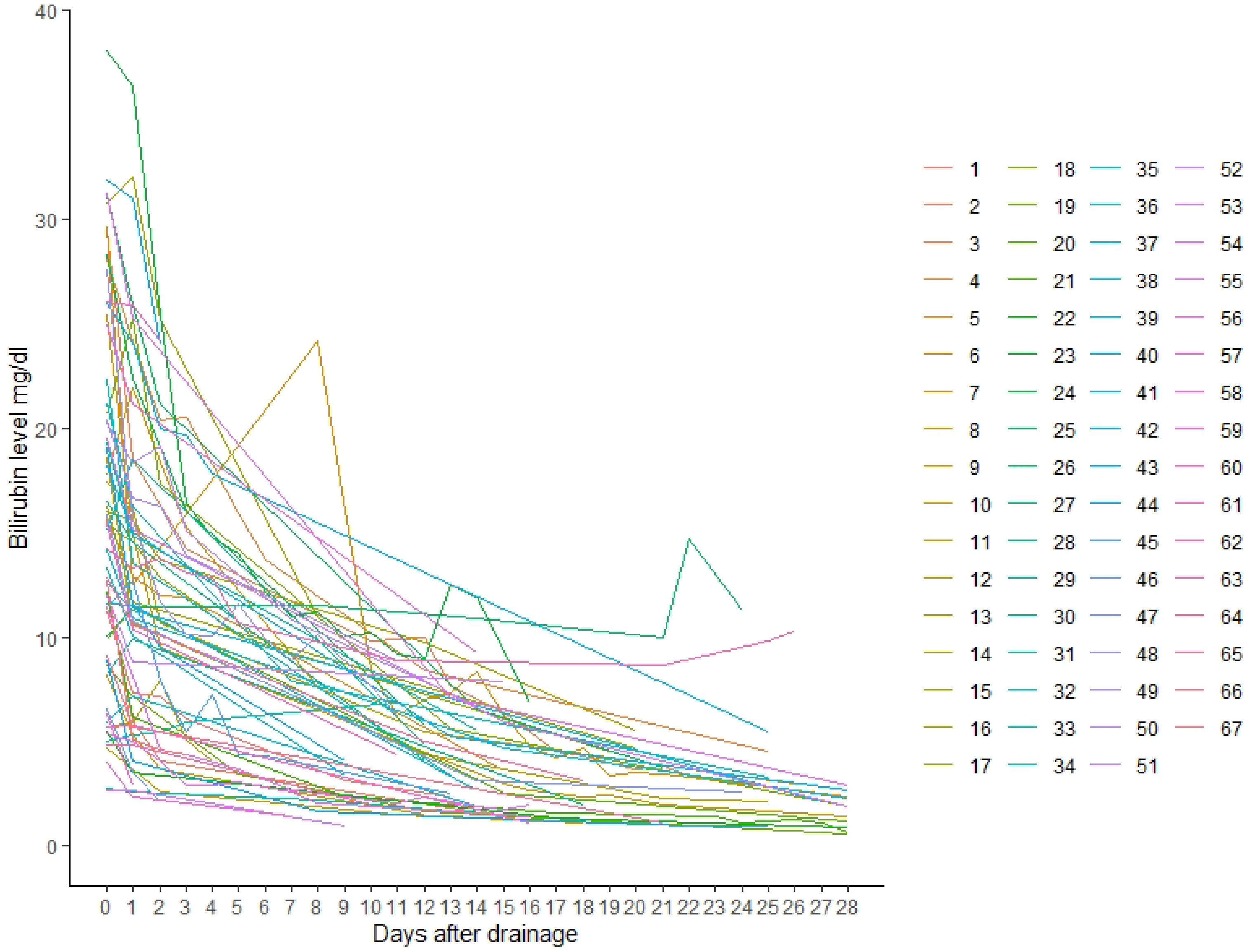

Table S1. Definitions of severe drainage-related complications.

Complication	Criteria
Cholangitis	Elevation in temperature ≥ 38,5°C and Leukocytes ≥10 $*10^9$/L, thought to have a biliary cause and requiring invasive intervention, without concomitant evidence of acute cholecystitis.
Acute cholecystitis	Radiologic evidence of cholecystitis, elevation in temperature more than 38.5°C and Leukocytes ≥10 $*10^9$/L, requiring emergency cholecystectomy or percutaneous drainage.
Persistent jaundice	Persistent elevated bilirubin levels after therapeutic success had initially been obtained, without signs of cholangitis or cholecystitis, requiring a reintervention.
Acute pancreatitis	Two or more of the following criteria be met for the diagnosis of acute pancreatitis: abdominal pain suggestive of pancreatitis, serum amylase or lipase level greater than three times the upper normal value, or characteristic imaging findings.
Biliary leak	Symptomatic intra-abdominal bile leakage due to bile duct perforation or leakage from the puncture tract documented by any radiographic technique requiring intervention.
Hemorrhage	Clinical evidence of bleeding requiring blood transfusion or reintervention.
